# Claudin-3-deficient C57BL/6J mice display intact brain barriers

**DOI:** 10.1038/s41598-018-36731-3

**Published:** 2019-01-18

**Authors:** Mariana Castro Dias, Caroline Coisne, Ivana Lazarevic, Pascale Baden, Masaki Hata, Noriko Iwamoto, David Miguel Ferreira Francisco, Michael Vanlandewijck, Liqun He, Felix A. Baier, Deborah Stroka, Rémy Bruggmann, Ruth Lyck, Gaby Enzmann, Urban Deutsch, Christer Betsholtz, Mikio Furuse, Shoichiro Tsukita, Britta Engelhardt

**Affiliations:** 1 Theodor Kocher Institute, University of Bern, Bern, Switzerland; 2 Laboratory of Tumor Immunology and Cell Therapy, Hyogo College of Medicine, Nishinomiya, Hyogo, Japan; 3 Division of Cell Biology, Kobe University Graduate School of Medicine, Kobe, Hyogo, Japan; 4 Interfaculty Bioinformatics Unit and Swiss Institute of Bioinformatics, University of Bern, Bern, Switzerland; 5 Karolinska Institutet/AstraZeneca Integrated Cardio Metabolic Centre (KI/AZ ICMC), Huddinge, Sweden; 6 Visceral Surgery Research Laboratory, Department of Biomedical Research, University of Bern, Bern, Switzerland; 7 Department of Immunology, Genetics and Pathology, Rudbeck Laboratory, Uppsala University, Uppsala, Sweden; 8 Division of Cell Structure, National Institute for Physiological Sciences, Okazaki, Japan; 9 Department of Physiological Sciences, School of Life Science, Okazaki, Japan; 10 Department of Cell Biology, Kyoto University Faculty of Medicine, Kyoto, Japan

**Keywords:** Tight junctions, Blood-brain barrier

## Abstract

The tight junction protein claudin-3 has been identified as a transcriptional target of the Wnt/β-catenin signaling pathway regulating blood-brain barrier (BBB) maturation. In neurological disorders loss of claudin-3 immunostaining is observed at the compromised BBB and blood-cerebrospinal fluid barrier (BCSFB). Although these observations support a central role of claudin-3 in regulating brain barriers’ tight junction integrity, expression of claudin-3 at the brain barriers has remained a matter of debate. This prompted us to establish claudin-3^−/−^ C57BL/6J mice to study the role of claudin-3 in brain barrier integrity in health and neuroinflammation. Bulk and single cell RNA sequencing and direct comparative qRT-PCR analysis of brain microvascular samples from WT and claudin-3^−/−^ mice show beyond doubt that brain endothelial cells do not express claudin-3 mRNA. Detection of claudin-3 protein at the BBB *in vivo* and *in vitro* is rather due to junctional reactivity of anti-claudin-3 antibodies to an unknown antigen still detected in claudin-3^−/−^ brain endothelium. We confirm expression and junctional localization of claudin-3 at the BCSFB of the choroid plexus. Our study clarifies that claudin-3 is not expressed at the BBB and shows that absence of claudin-3 does not impair brain barrier function during health and neuroinflammation in C57BL/6J mice.

## Introduction

Homeostasis of the central nervous system (CNS) is preserved by the blood-brain barrier (BBB) and the blood-cerebrospinal-fluid barrier (BCSFB), by creating a separation between the CNS and the bloodstream and thus protecting the CNS from infectious and toxic agents. Barrier function at the BBB is established at the level of highly specialized microvascular endothelial cells, whereas the BCSFB is established by the choroid plexus epithelium^1^. Under physiological conditions, the brain barriers control transcellular and paracellular passage of molecules and solutes in and out of the CNS by the presence of complex and continuous tight junctions (TJs)^2,3^. The integral membrane proteins found to localize to TJs are the junctional adhesion molecules (JAM), occludin and the members of the claudin family^1^. Claudins are integral 4-pass transmembrane proteins exclusively located at TJs and in contrast to both JAMs and occludin, are sufficient for TJs induction^4^. In mammals, the claudin family is composed of 27 known members that display tissue specific expression patterns and different functions. While some claudins, e.g. claudin-1 and claudin-3 form paracellular barriers, other claudins, e.g. claudin-2 or claudin-16, form paracellular pores allowing for controlled diffusion of ions and water via the TJs^5^. Each TJ is established by a combination of different claudins and therefore the tightness of individual strands of TJs is determined by the combination and mixing ratio of claudins^6^. At their C-terminus claudins have a PDZ-binding motif, which mediates their interaction with the intracellular scaffolding proteins ZO-1, ZO-2 and ZO-3 linking the claudins to the actin cytoskeleton^7^.

Claudin-5 is an endothelial cell-specific component of TJ strands and it is highly expressed in BBB TJs of rodents, zebrafish, nonhuman primates and humans^8–10^. Claudin-5 forms a paracellular barrier as its constitutive lack leads to perinatal death in mice due to the uncontrolled diffusion of small molecules across BBB TJs^8^ and induced suppression of claudin-5 in adult mice leads to seizures and death^11^. Additional claudins reported to be present in BBB TJs are claudin-3 and claudin-12 with their precise functions in BBB TJs to be determined^12–15^. TJs of the BCSFB have been reported to be composed of claudin-1, -2, -3 and -11^16–18^. With claudin-1 forming a paracellular barrier and claudin-2 forming a paracellular water channel, BCSFB TJs may be adapted to the role of the choroid plexus in producing cerebrospinal fluid (CSF)^19–21^. Finally, claudin-11 is responsible for the induction of the unique parallel TJ strands observed in choroid plexus epithelial cells^16^.

BBB dysfunction is correlated with several neurological disorders including multiple sclerosis (MS) and detected in patients as gadolinium-enhancing lesions in magnetic resonance imaging^22^. BBB impairment is correlated with alterations of the junctional complexes of the BBB^23,24^ thus reinforcing the notion that TJ breakdown contributes to BBB dysfunction in MS^23–25^. In addition, there is accumulating evidence for an involvement of the choroid plexus in neurological disorders including MS^26–28^. However, little is known about specific alterations in the junctional architecture of the BCSFB under neuroinflammatory conditions^16,29^.

Experimental autoimmune encephalomyelitis (EAE), an animal model for MS, recapitulates the changes in TJs architecture observed in MS^30^. A specific role for claudin-3 in establishing and maintaining BBB and BCSFB TJ integrity has been suggested by a number of studies. In EAE, junctional immunostaining for claudin-3 is selectively lost from inflamed CNS microvessels surrounded by infiltrating immune cells^12^. Junctional claudin-3 immunostaining is also lost in the BCSFB of the choroid plexus of MS patients^29^. Additional evidence for a role of claudin-3 in brain barrier integrity is derived from its identification as a downstream effector of the Wnt/β-catenin signaling pathway for BBB maturation during embryogenesis^31^. At the same time, expression of claudin-3 at the BBB TJs has repeatedly been questioned by others^18,32^.

To clarify expression and function of claudin-3 in the TJs of the brain barriers, we established claudin-3^−/−^ mice and backcrossed them to the C57BL/6 background allowing to study brain barrier function in health and neuroinflammation in EAE. Unexpectedly, we observed and validated that claudin-3 is not expressed in TJs of mouse BBB endothelial cells, *in vitro* and *in vivo*. Prior evidence supporting claudin-3 expression at the BBB is due to cross-reactivity of anti-claudin-3 antibodies with an unknown endothelial junctional antigen detectable in BBB TJs of claudin-3^−/−^ mice. We confirmed presence of claudin-3 in the epithelial TJs of the BCSFB. Notably, in C57BL/6 mice lacking claudin-3 we observed no impairment of the brain barrier properties *in vivo*.

## Results

### Generation and characterization of claudin-3^−/−^ C57BL/6J mice

**Figure 1:**
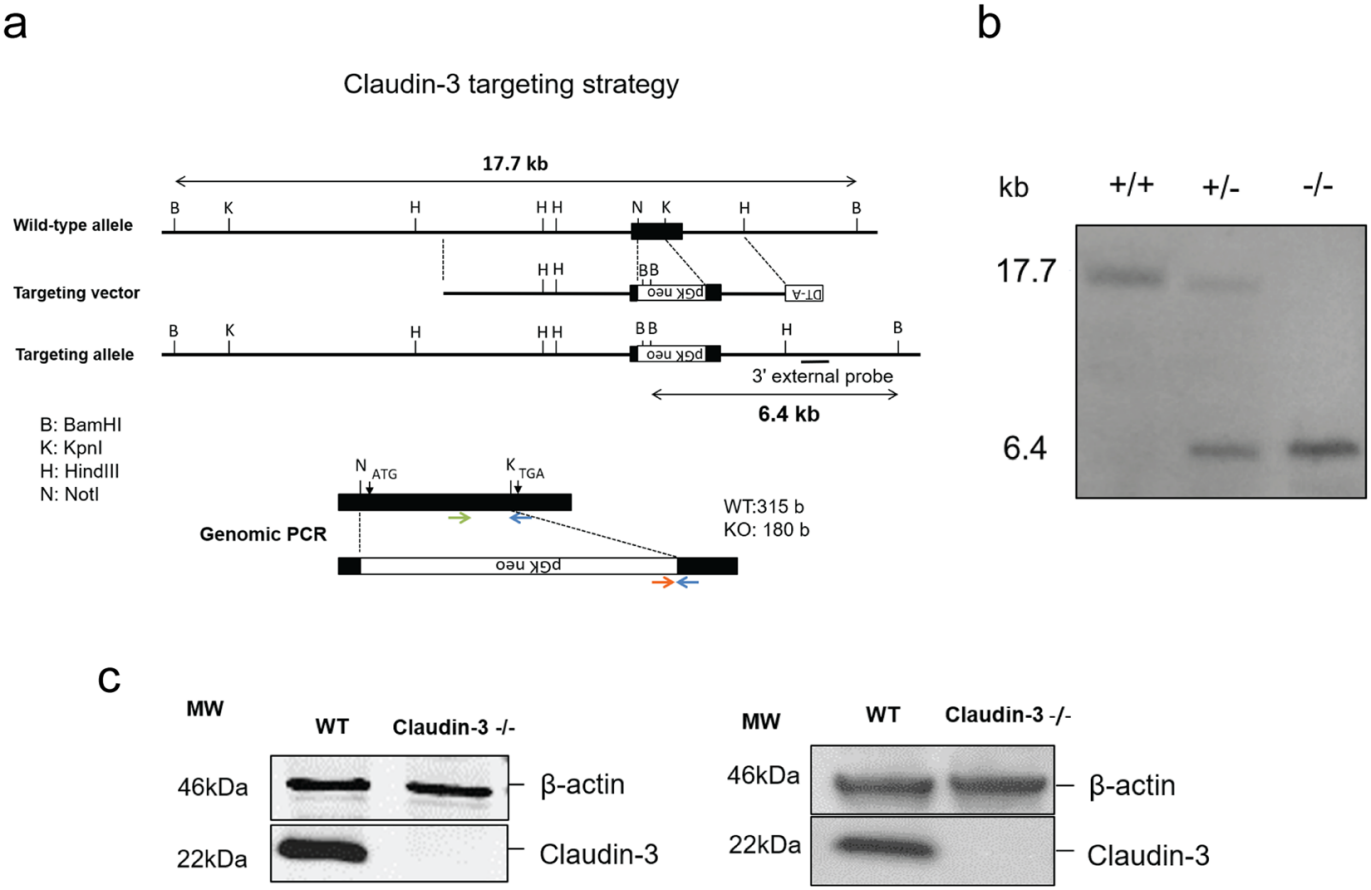
Claudin-3 targeting strategy in C57BL/6J mice. (**a**) Schematic representation of the KO strategy. Restriction sites are indicated for the WT allele, the targeting vector and the targeted allele of the mouse claudin-3 gene. The open reading frame of claudin-3 is encoded by a single exon. In the targeted allele, a large part of the exon encoding amino acids 1–207 of claudin-3 is replaced by a PGK-neo cassette. The position of the 3′ probe for Southern blotting is indicated as a bar. B, BamHI; K,KpnI; H, HindIII; N, NotI. (**b**) Southern Blot of genomic DNA isolated from spleens of WT, claudin-3^+/−^ and claudin-3^−/−^ C57BL/6J mice, with a BamHI digestion. Southern blotting with the probe indicated in **a** yielded a 17.7- and 6.4-kb band from the WT and targeted allele, respectively. (**c**) Loss of claudin-3 protein examined by immunoblot analysis with an anti-claudin-3 polyclonal antibody (Novus Biologicals). Freshly isolated choroid plexus (left) and liver (right) samples from 10 WT and 10 claudin-3^−/−^ C57BL/6J mice were pooled per sample. The cropped blots are shown in this figure and the full-length blots are presented in Supplementary Fig. S7. In total, three independent WT and claudin-3^−/−^ choroid plexus or liver samples were analyzed.

A knock-out (KO) allele of the mouse claudin-3 was created by gene targeting in embryonic stem cells (ES). The targeting vector was designed to replace most of the coding region of claudin-3 by insertion of a PGK neo cassette except for the last 30 nucleotides of the open reading frame precluding expression of any truncated claudin-3 peptide (Fig. 1a). Correct gene targeting in ES cells and germline transmission were confirmed by Southern blotting (Fig. 1b) and absence of claudin-3 protein in claudin-3−/− C57BL/6J mice was confirmed by Western blotting and immunofluorescence staining (Fig. 1c and Supplementary Fig. S5a). Homozygous mutant mice were created by interbreeding heterozygous parents. Homozygous claudin-3^−/−^ mice survive to weaning age at close to Mendelian ratios (23.5%) (Supplementary Table S1). Adult mutant mice are phenotypically normal, fertile and healthy and were backcrossed for more than 10 generations to the C57BL/6J background allowing for reproducible *in vivo* experimentation on a homogeneous genetic background.

### Claudin-3 is not expressed in mouse brain endothelial cells *in vitro*

**Figure 2:**
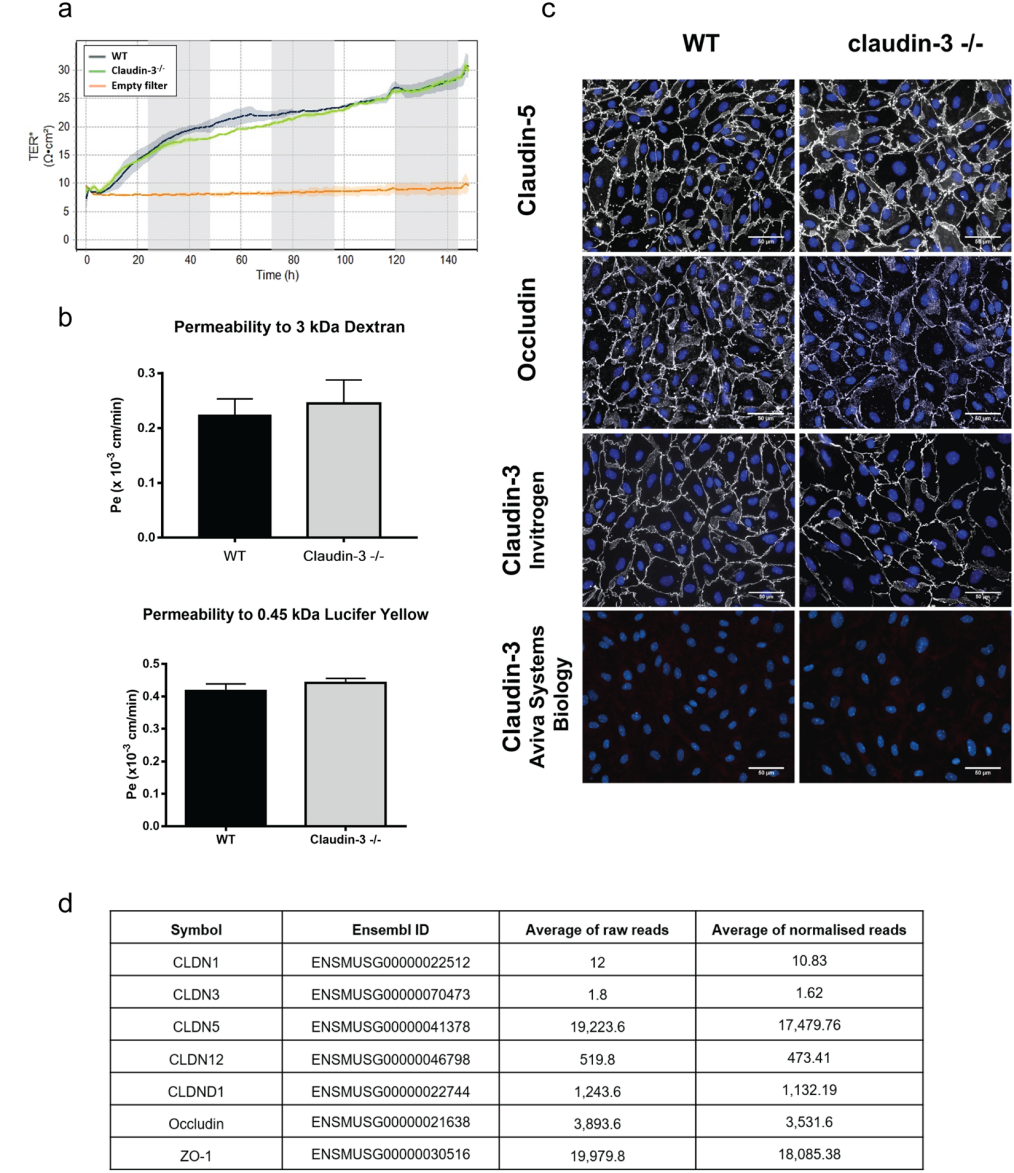
Claudin-3 is not expressed in primary mouse brain microvascular endothelial cells *in vitro*. (**a**) The time-dependent progression of the TEER of pMBMECs isolated from WT (blue curve) and claudin-3^−/−^ C57BL/6J mice (green curve) and grown on 0.4 μm pore size insert filters was measured by impedance spectroscopy using the cellZscope device. Graphs show a period of 160 hours from day 1 to day 7 after plating of pMBMECs. Lines and shades represent means of four filters, with the SEM shown as shaded area. The orange line shows TEER across matrigel-coated empty filters. The graph is representative of three independent experiments. (**b**) Permeability for 3 kDa Dextran and 0.45 kDa Lucifer Yellow of WT and claudin-3^−/−^ pMBMEC monolayers was measured at day 7 of culture. Endothelial permeability coeficient (P_e_) values were calculated based on measuring diffusion of tracers across pMBMEC monolayers at four sequential time points with 10 min intervals exactly as described before^34^. Bars show mean P_e_ ± SD as calculated from three independent experiments analysing a total of 14 filters per condition for Dextran and 19 filters for Lucifer Yellow. (**c**) Comparable junctional immunofluorescence staining for claudin-5 and occludin is detected in monolayers of WT and claudin-3^−/−^ pMBMECs. Anti-claudin-3 antibodies either show junctional immunostaining or no immunostaining of WT and claudin-3^−/−^ pMBMEC monolayers. Scale bar = 50 μm. (**d**) RNAseq analysis of pMBMECs. Average of raw reads and corresponding normalised values are shown for claudin-1 (CLDN1), claudin-3 (CLDN3), claudin-5 (CLDN5), claudin-12 (CLDN12), claudin-like-domain-containing-1 (CLDND1), occludin and ZO-1, from five independent pMBMEC samples pooled from 10 mice each. A threshold of 100 was established for the normalized reads, above which all transcripts were considered as expressed.

To study how absence of claudin-3 impairs barrier characteristics of the BBB we first made use of our well characterized *in vitro* model of the mouse BBB, in which freshly isolated primary mouse brain microvascular endothelial cells (pMBMECs) retain mature BBB TJs, express BBB specific transporters and display high transendothelial electrical resistance (TEER) and low permeability to small molecular tracers^33,34^. Impedance spectroscopy showed that pMBMECs isolated from claudin-3^−/−^ and wild-type (WT) C57BL/6J mice displayed comparable kinetics in establishing comparable TEER across the pMBMEC monolayers (Fig. 2a). Similarly, diffusion of the small molecular tracers, 3 kDa Dextran and 0.45 kDa Lucifer Yellow, showed no difference between the pMBMEC monolayers established from claudin-3^−/−^ and WT C57BL/6J mice (Fig. 2b). In parallel, immunofluorescence stainings for TJ proteins on pMBMEC monolayers from claudin-3^−/−^ and WT C57BL/6J mice showed no difference in the junctional localization of claudin-5, occludin, ZO-1, ZO-2, JAM-A, VE-cadherin and β-catenin between claudin-3^−/−^ and WT pMBMEC monolayers (Fig. 2c and Supplementary Fig. S1). Unexpectedly, we observed junctional immunostainings for claudin-3 in WT and in claudin-3^−/−^ pMBMEC monolayers when employing a polyclonal anti-claudin-3 antibody (Invitrogen) (Fig. 2c). Recognition of this reagent of mouse claudin-3 and lack of cross-reactivity with claudin-1 and claudin-5 was confirmed by immunofluorescence staining and Western blotting of claudin transfectants (Supplementary Table S2). At the same time a second polyclonal anti-claudin-3 antibody (Aviva Biology Systems) failed to show positive immunostaining for claudin-3 on both, WT and claudin-3^−/−^ pMBMEC monolayers (Fig. 2c). We therefore reasoned that conventional immunization protocols may fail to produce non-cross reacting anti-claudin-3 antibodies due to the highly conserved nature of claudins. Thus, we chose a genetic immunization approach in claudin-3^−/−^ C57BL/6J mice against the lacking gene product as this approach has the potential to yield a wide range of antibody reactivities targeting the extracellular domains of claudin-3 across species boundaries^35^. Although this approach produced monoclonal mouse-anti-mouse claudin-3 antibodies detecting extracellular domains of claudin-3 in L-cell transfectants, none of the antibodies detected claudin-3 on cultured pMBMECs or cultured primary mouse choroid plexus epithelial cells or in unfixed frozen mouse brain sections (Supplementary Fig. S2 and data not shown). Thus, reliable detection of claudin-3 protein in pMBMECs was not possible.

We therefore decided to profile the transcriptome of pMBMECs by RNA sequencing (RNAseq). Analyzing five different samples of pMBMECs, we confirmed high expression of claudin-5 in pMBMECs by detection of raw counts of reads between 16,588 to 22,205 (raw average of 19,223.6; post normalization average value 17,479.76); expression of claudin-12 with raw counts of reads between 455 and 591 (raw average 519.8 and post-normalization average value 473.41) (Fig. 2d). We confirmed absence of claudin-1^36^, with a detection of raw counts of reads varying from 8 to 15 (raw average of 12 and a post-normalization average value 10.83), which was below 100, the threshold for considered expression. Surprisingly, raw counts of reads for claudin-3 ranged from 0 to 6 reads in the five pMBMECs samples investigated, amounting to an average of 1.8 for raw counts and an average of approximately 1.62 for the normalized values (Fig. 2d). Thus, RNAseq has allowed to unequivocally demonstrate that pMBMECs do not express claudin-3 mRNA. Junctional immunostaining in WT and claudin-3^−/−^ pMBMECs with anti-claudin-3 reagents (Fig. 2c) must thus be due to cross-reaction with a junctional antigen different from claudin-3, and present in claudin-3^−/−^ pMBMECs. False positive detection of junctional claudin-3 protein in pMBMECs was not due to antibody cross-reactivity with claudin-5 or CLDND1, both found to be expressed in pMBMECs by RNAseq (Fig. 2d, Supplementary Table S2 and data not shown).

### Claudin-3 is not expressed in mouse brain endothelial cells *in vivo*

**Figure 3:**
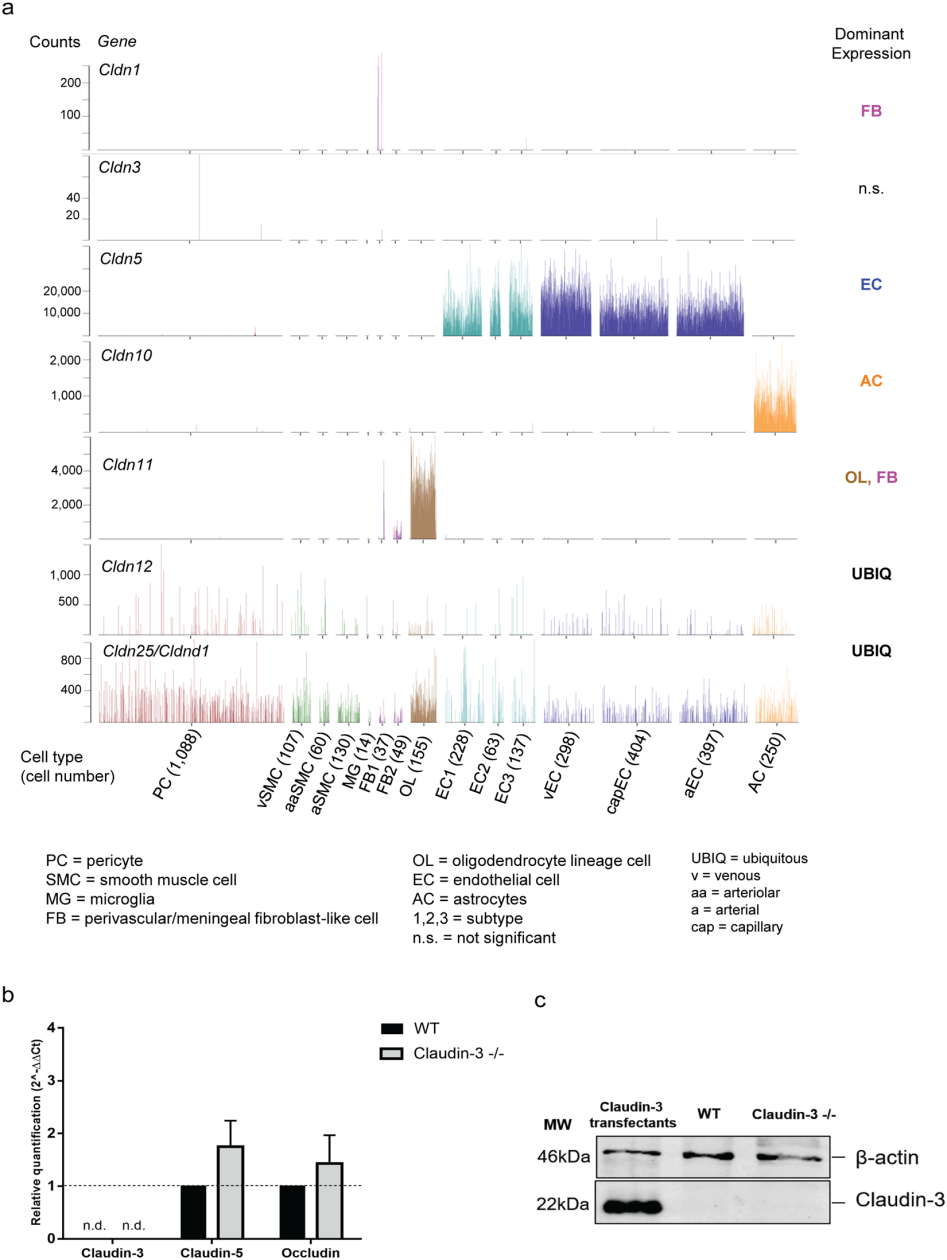
Claudin-3 is not expressed in mouse brain endothelial cells *ex vivo*. (**a**) Expression of claudins at the BBB depicted by single cell RNA sequencing. The bar-plots shown are excerpts from http://betsholtzlab.org/VascularSingleCells/database.html ^37,38^ and represent the claudins that show the most significant levels of expression in any of the cell types comprising the brain vasculature (EC, PC, SMC) or vessel-associated cell types such as FB, MG, AC and OL. Cell type abbreviations are provided at the bottom of the figure along with the cell numbers of each type in between brackets. The dominant cell-type specific expression for each of the shown claudins is indicated to the right. (**b**) Relative gene expression of claudin-3, claudin-5 and occludin in samples from freshly isolated brain microvessels from WT and claudin-3^−/−^ C57BL/6J mice was assessed by qRT-PCR. For each gene, technical triplicates in four independent experiments were measured. Relative quantification is represented by the 2^−ΔΔCt^ value. N.d. stands for not detectable. (**c**) Immunoblot analysis of claudin-3 protein levels in freshly isolated brain microvessels from WT and claudin-3^−/−^ C57BL/6J mice using a polyclonal anti-claudin-3 antibody (Novus Biologicals). Lysates of claudin-3 L-cells transfectants were used as positive controls. A cropped blot is shown in this figure and the full-length blot is presented in Supplementary Fig. S7. Three independent brain microvessel samples pooled from 10 WT and 10 claudin-3^−/−^ C57BL/6J mice were analyzed.

To determine if claudin-3 is expressed in brain endothelial cells *in vivo*, we next took advantage of our recent single cell RNA sequencing dataset comprising the analysis of more than 1,500 endothelial cells and 1,000 pericytes, in addition to data from another ≈1,000 cells, including vascular smooth muscle cells, astrocytes, oligodendrocytes, microglia and perivascular and meningeal fibroblast-like cells of the adult mouse brain^37,38^. For each cell type, our dataset provides comprehensive, genome-wide and quantitative transcriptional information. We found RNA sequences from 21 different claudins (Fig. 3a), including claudin-3. However, for 13/21 of the claudins, RNA sequences were found in only a few (<10) individual cells and without bias for cell type and were hence considered background noise. One of these was claudin-3, for which sequences were obtained from only 4 cells, one of which clustered as endothelial cell. The remaining ≈1,500 endothelial cells were claudin-3 negative (Fig. 3a). In marked contrast, 8 of the claudins displayed significant expression, many of them in a cell-type specific fashion. Of all claudins, only claudin-5 was endothelial specific. Claudin-12 and CLDND1 (referred as claudin-25 in this dataset) were also found in endothelial cells, albeit at much lower levels than claudin-5 but lacked cell type specificity and were instead expressed across all cell types in relatively uniform levels with few exceptions (Fig. 3a). Besides claudin-5, five other claudins displayed striking cell type-specific expression: claudin-10 in astrocytes, claudin-11 and claudin-14 in oligodendrocytes, claudin-15 in pericytes and claudin-1 in a subpopulation of fibroblast-like cells (Fig. 3a and Supplementary Fig. S3). Our data provide compelling evidence that claudin-3 is neither expressed in brain endothelial cells nor in any other perivascular or glial cell types in adult mice.

Direct comparison of claudin-3 expression in freshly isolated brain microvessels from WT and claudin-3^−/−^ C57BL/6J mice by qRT-PCR confirmed absence of claudin-3 mRNA expression in brain endothelial cells *ex vivo*, with relative expression levels for claudin-3 found indistinguishable between brain vascular samples from WT and claudin-3^−/−^ C57BL/6J mice (Fig. 3b). At the same time, expression of both claudin-5 and occludin could be readily detected at equal levels in microvessels freshly isolated from the brains of WT and claudin-3^−/−^ C57BL/6J mice (Fig. 3b). qRT-PCR confirmed negligible contamination of these microvascular samples with choroid plexus epithelial cells, since we observed insignificant expression of claudin-1, E-cadherin and transthyretin (Ttr) in the brain microvessel preparations (Supplementary Table S3). Thus, the availability of claudin-3^−/−^ mice allows to demonstrate beyond a doubt that mouse brain microvascular endothelial cells do not express claudin-3.

Considering these unexpected findings, we next explored if absence of claudin-3 mRNA from mouse brain microvascular endothelial cells could be reconciled with earlier reports demonstrating the presence of claudin-3 protein *in vivo* by immunodetection. To this end, we first performed Western blots of samples from freshly isolated highly purified brain microvessels of WT and claudin-3^−/−^ C57BL/6J mice and of lysates of cultured claudin-3 transfectants as positive control. Taking into account the detection of an unknown cross-reacting protein by some anti-claudin-3 antibodies when performing immunofluorescence staining on claudin-3 deficient specimen, Western blot analysis was also performed with a different anti-claudin-3 antibody (Novus Biologicals), which allowed to detect a 22 kDa band for claudin-3 in claudin-3 transfectants but not in samples of freshly isolated brain microvessels from WT and claudin-3^−/−^ C57BL/6J mice (Fig. 3c), underscoring the absence of claudin-3 protein from brain endothelial cells and confirming the mRNA data.

**Figure 4:**
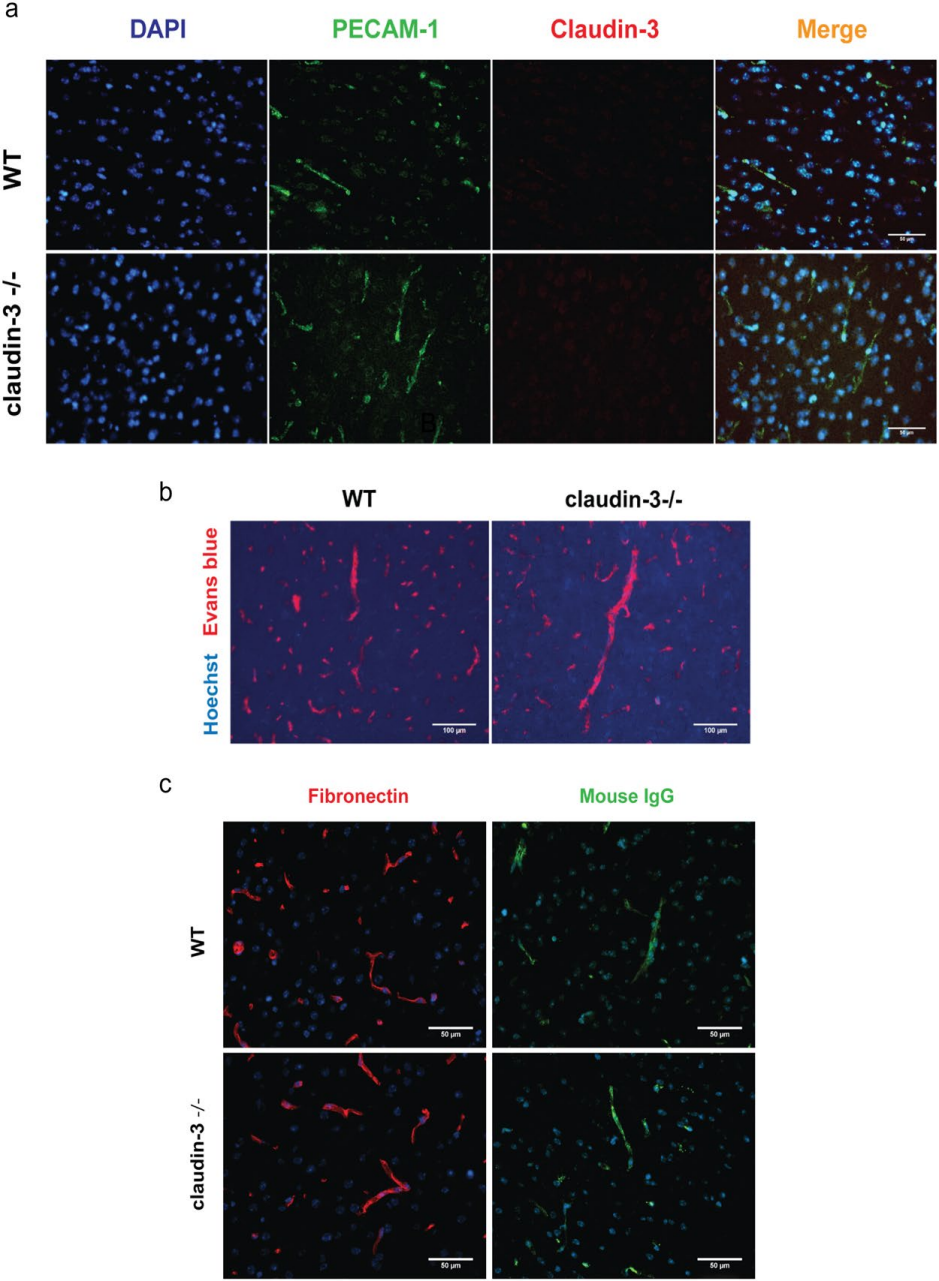
Lack of claudin-3 expression in mouse brain endothelial cells *in vivo* does not impair BBB integrity. (**a**) Immunofluorescence staining of frozen brain sections from WT and claudin-3^−/−^ C57BL/6J mice for PECAM-1 (green) and claudin-3 (red). Nuclei are stained with DAPI (blue). Anti-claudin-3 antibody from Novus Biologicals does not stain brain endothelial cells in WT and claudin-3^−/−^ C57BL/6J mice. Three independent stainings were done. Scale bar = 50 μm. (**b**,**c**) *In vivo* BBB permeability to i.v. injected exogenous tracers Hoechst and Evans blue (scale bar = 100 μm) (**b**) and to the endogenous plasma tracers fibronectin and murine IgG (scale bar = 50 μm) (**c**), in frozen brain sections of WT and claudin-3^−/−^ C57BL/6J mice is shown. Brain cryosections from two mice per genotype were analyzed in two independent experiments.

As we and others have previously observed junctional immunostaining for claudin-3 in microvessels in brain sections of mice^12,31^, we finally investigated localization of claudin-3 protein in frozen brain sections of WT and claudin-3^−/−^ C57BL/6J mice by immunofluorescence staining. Notably, using several anti-claudin-3 antibodies we detected junctional immunostaining in brain microvessels in sections from claudin-3^−/−^ C57BL/6J mice (Supplementary Fig. S4). Importantly, anti-claudin-3 antibodies that did not show any endothelial immunostaining in brain sections from claudin-3^−/−^ C57BL/6J mice, also never produced any immunostainings in microvessels on frozen brain sections from WT C57BL/6J mice (Fig. 4a and Supplementary Table S2), underscoring the absence of claudin-3 also at the protein level in brain endothelial cells *in vivo*.

Functional evidence for the lack of any role for claudin-3 in BBB integrity was finally obtained by investigating the *in vivo* permeability for exogenous (Evans blue and Hoechst, 3 kDa and 10 kDa Dextran) and endogenous vascular tracers (fibronectin and mouse IgG) across the BBB in WT and claudin-3^−/−^ C57BL/6J mice by immunofluorescence staining of brain sections. While all tracers were readily detected in the choroid plexus stroma, where they could pass the fenestrated microvessels, none of the tracers was found to penetrate the BBB in brain sections of WT nor in claudin-3^−/−^ C57BL/6J mice (Fig. 4b,c and data not shown).

Taken together, combining RNAseq of single brain endothelial cells and direct comparison of brain microvessel samples of WT and claudin-3^−/−^ C57BL/6J mice by qRT-PCR and Western blotting has allowed to unequivocally show that mouse brain endothelial cells do not express claudin-3. Furthermore, anti-claudin-3 antibodies displaying no immunoreactivity with brain endothelial cells in tissue samples from claudin-3^−/−^ C57BL/6J mice confirmed absence of claudin-3 in brain endothelial cells of WT mice *in vivo*. Naturally, claudin-3^−/−^ C57BL/6J mice display intact BBB integrity *in vivo*.

### Absence of claudin-3 does not impair integrity of the BCSFB

**Figure 5:**
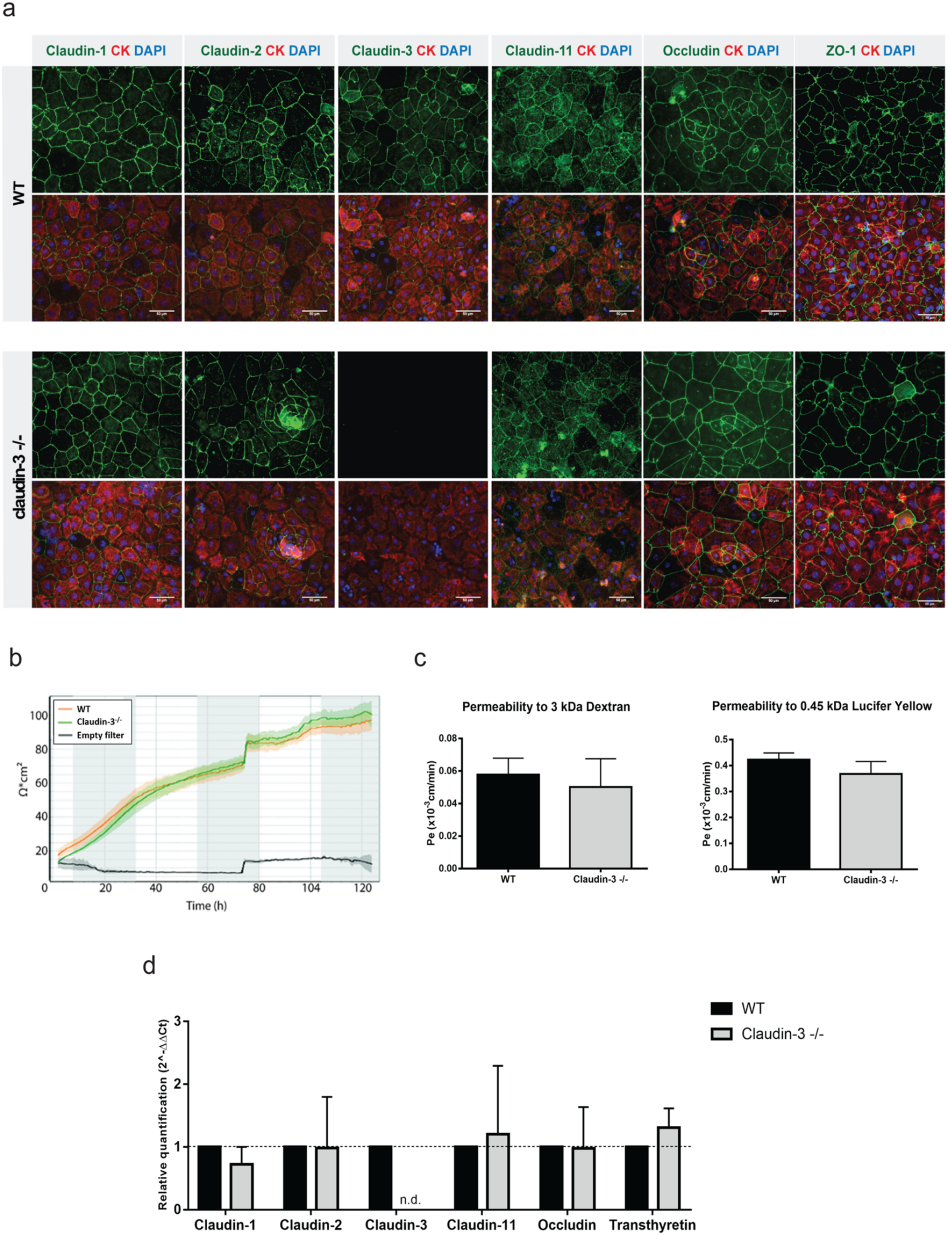
Lack of claudin-3 does not impair BCSFB integrity *in vitro*. (**a**) Tight junction protein localization in pMCPEC monolayers established from WT and claudin-3^−/−^ C57BL/6J mice was analyzed by immunofluorescence staining. Upper row of images shows junctional staining for claudins-1, -2, -3, -11, occludin and ZO-1 on WT and for claudin-1, -2, -11, occludin and ZO-1 on claudin-3^−/−^ pMCPEC monolayers (green), respectively. Lower row of images shows the merge of the green junctional stainings of pMCPECs with a positive staining for cytokeratin (CK, red) and the nuclei (DAPI in blue). Stainings were performed in three independent experiments. Scale bar = 50 μm. (**b**) The time-dependent progression of the TEER of pMCPEC monolayers isolated from WT (orange curve) and claudin-3^−/−^ C57BL/6J mice (green curve) is shown over a period of 280 hours from day 1 to day 7 after plating of pMCPECs on 0.4 μm pore size insert filters. Lines and shades represent means of four filters, with the SEM shown as shaded area. The blue line shows TEER across laminin coated empty filters. Data were assessed by impedance spectroscopy using the cellZscope device and the graph is representative for four independent experiments. (**c**) Permeability for 3 kDa Dextran and 0.45 kDa Lucifer Yellow of WT and claudin-3^−/−^ pMCPEC monolayers was measured at day 7 of culture. Epithelial permeability coefficient (P_e_) values were calculated based on measuring diffusion of tracers across pMCPEC monolayers at four sequential time points with 10 min intervals exactly as described before^34^. Bars show the mean permeability coefficients P_e_ ± SD of five independent experiments with three filters per condition. (**d**) Relative gene expression of claudin-1, -2, -3, -11, occludin and transthyretin (positive control) in samples of pMCPECs established from WT and claudin-3^−/−^ C57BL/6J mice, after seven days in culture, and assessed by qRT-PCR. For each gene, technical triplicates in five independent experiments were measured. Relative quantification is represented by the 2^−ΔΔCt^ value. N.d. stands for not detectable.

In addition to the BBB, claudin-3 has also been described to localize to TJs of the BCSFB in the choroid plexus^39^. To verify localization of claudin-3 in choroid plexus epithelial cells, we first made use of our *in vitro* model of the BCSFB, in which freshly isolated primary mouse choroid plexus epithelial cells (pMCPECs) retain phenotypic properties of the BCSFB *in vivo* including junctional localization of TJ proteins, high TEER and low permeability to small molecular tracers^40^. Immunostaining for claudin-3 in cytokeratin^+^ pMCPECs from WT and claudin-3^−/−^ C57BL/6J mice showed junctional immunostaining for claudin-3 protein in pMCPEC monolayers established from WT but not from claudin-3^−/−^ mice with all polyclonal antibodies employed (Fig. 5a), irrespective of their production of a junctional immunostaining in brain microvessels of claudin-3^−/−^mice (Supplementary Fig. S4). This confirmed junctional localization of claudin-3 protein at the BCSFB *in vitro*. Immunostainings for claudin-3 on frozen brain sections exposing the choroid plexuses of the 3^rd^, lateral or 4^th^ ventricle of WT and claudin-3^−/−^ C57BL/6J mice produced for some antibodies junctional immunostaining of choroid plexus epithelial cells in brain sections from WT but not from claudin-3^−/−^ C57BL/6J mice (Supplementary Fig. S5), while other antibodies completely failed to display any specific immunostaining of choroid plexus epithelial cells in brain sections from both, WT and claudin-3^−/−^ C57BL/6J mice (Supplementary Table S2). Unlike observed for brain endothelial cells, we did not observe junctional immunostaining with any of the anti-claudin-3 antibodies at the BCSFB in brain sections from claudin-3^−/−^ C57BL/6J mice. Taken together, these observations confirm junctional localization of claudin-3 at the BCSFB in the choroid plexus. Importantly, these data propose that the cross-reactive junctional epitope detected by some anti-claudin-3 antibodies in brain microvessels is not present in the choroid plexus.

To next explore if absence of claudin-3 has any influence on BCSFB integrity, immunostainings for claudin-1, -2, -11, occludin and ZO-1 (Fig. 5a) produced comparable junctional staining in pMCPEC monolayers from WT and claudin-3^−/−^ C57BL/6J mice. Thus, absence of claudin-3 did not lead to obvious alterations of the TJ architecture of the BCSFB *in vitro*. Impedance spectroscopy demonstrated that pMCPECs isolated from claudin-3^−/−^ and WT C57BL/6J mice displayed the same kinetics in establishing a high TEER across the pMCPEC monolayer (Fig. 5b). Similarly, diffusion of small molecular tracers, namely 3 kDa Dextran and 0.45 kDa Lucifer Yellow, across pMCPEC monolayers, showed no difference between claudin-3^−/−^ and WT C57BL/6J mice (Fig. 5c). Thus, absence of claudin-3 did not impair barrier characteristics of the BCSFB *in vitro*.

As absence of claudin-3 has been proposed to regulate BCSFB integrity^29^, we next asked if absence of claudin-3 would influence expression levels of other tight junction molecules in pMCPECs thus masking a potential role of claudin-3 in regulating *in vitro* BCSFB integrity. Direct comparative qRT-PCR analysis of samples from pMCPEC cultures of WT and claudin-3^−/−^ mice confirmed presence of claudin-3 mRNA in WT and its absence in claudin-3^−/−^ pMCPECs and showed no difference in mRNA expression of claudin-1, -2, -11 and occludin (Fig. 5d). Thus, absence of claudin-3 does not impair TJ characteristics of the BCSFB *in vitro*.

**Figure 6:**
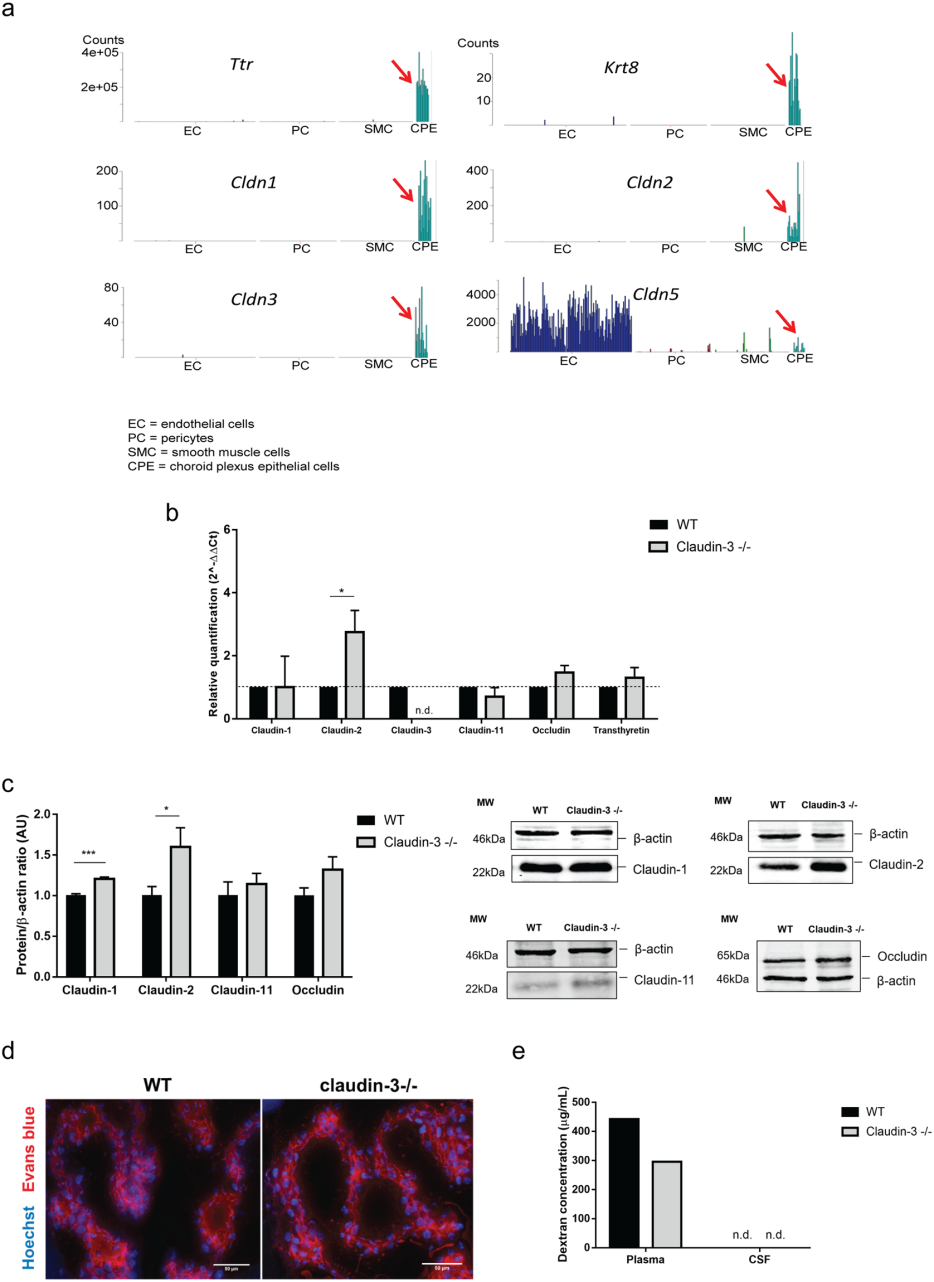
Absence of claudin-3 promotes up-regulation of tight junction proteins in the choroid plexus. (**a**) Expression of claudins in choroid plexus epithelial cells depicted by single cell RNA sequencing. The graphs are from a dataset distinct from that available at http://betsholtzlab.org/VascularSingleCells/database.html ^37,38^. In this dataset a small but distinct set of choroid plexus epithelial cells (CPE, indicated by red arrows) were identified based on the highly specific marker Ttr; note the order of magnitude of expression of this gene relative to all other genes. The identity of the CPE cluster is further supported by the expression of several cytokeratins, including *Krt8, Krt18* and *Krt23*. Claudins expression in the CPE cluster is shown, specifically for claudin-1, -2, -3 and -5. Note the differences in sequence counts (Y-axes) reflecting different levels of RNA expression. (**b**) Relative gene expression levels of claudin-1, -2, -3, -11, occludin and transthyretin (positive control) was performed on samples of freshly isolated choroid plexuses from WT and claudin-3^−/−^ C57BL/6J mice, assessed by qRT-PCR. For each gene, technical triplicates in five independent experiments were measured. Relative quantification is represented by the 2^−ΔΔCt^ value. N.d. stands for not detectable. (**c**) Immunoblot analysis for claudin-1, claudin-2, claudin-11 and occludin in samples of freshly isolated choroid plexuses pooled from 10 WT and claudin-3^−/−^ C57BL/6J mice, respectively. For each protein, three independent choroid plexus samples from WT and for claudin-3^−/−^ C57BL/6J mice were analysed. Cropped blots are shown in this figure and the full-length blots are presented in Supplementary Fig. S7. Bar graph shows mean ± SD of the performed independent experiments. Statistical analysis with unpaired t-test with Welch’s correction (*p < 0.05; ***p < 0.001). (**d**) *In vivo* BCSFB permeability to i.v. injected exogenous tracers Hoechst and Evans blue in frozen brain tissue sections of WT and claudin-3^−/−^ C57BL/6J mice is shown. Brain cryosections from two mice per genotype were analysed in two independent experiments. Scale bar = 50 μm. (**e**) *In vivo* permeability assay to intramuscular injected exogenous tracer 3 kDa Dextran-TexasRed to CSF of WT and claudin-3^−/−^ C57BL/6J mice is shown. CSF was pooled from 6 mice from each genotype and plasma was collected as a positive control. N.d. stands for not detected.

To finally confirm claudin-3 expression at the BCSFB of the choroid plexus *in vivo*, we made use of a previously unpublished single cell RNAseq data set, in which we used a slightly different protocol for brain tissue dissociation than above^37^, leading to a proportion of cells that were not fully dissociated into single cells and therefore gave rise to mixed transcriptomes (Fig. 6a). In our experience^37^, this is a particular problem for endothelial cells and pericytes, which are embedded within a common basement membrane, and as a result sorted endothelial cells often carry pieces of pericytes and *vice versa*. In the experiment shown in Fig. 6a, we sorted 2 × 384 cells from *Pdgfrb-GFP:Cspg4-dsRed* aiming for pericytes^41^ and 384 cells from *claudin-5-GFP* mice aiming for endothelial cells. After quality filtering, 942 cells remained. *t*-SNE analysis showed expected clusters of endothelial cells and mural cells (pericytes and vSMC), but in addition, some of the cells sorted from *Pdgfrb-GFP:Cspg4-dsRed* mice formed a distinct cluster containing choroid plexus epithelial cells, as identified using the canonical CP cytokeratins (Krt8, Krt18, Krt23) and Ttr as markers. Apparently, these cells were sorted because they were attached to (pieces of) pericytes expressing *Pdgfrb* and *Cspg4*. Following clustering by BackSPIN^42^ and expression analysis using barplots, the major patterns of claudin expression in endothelial cells and pericytes shown above (Fig. 3a and Supplementary Fig. S3) were confirmed. However, the choroid plexus epithelial cluster displayed highly specific expression of several claudins (1,2,3 and 6) as well as the ubiquitously expressed CLDND1 (referred as claudin-25 in this dataset) (Fig. 6a). Claudin-9, -10, -12 and -22 were also detected to be expressed in these epithelial cells but are not specific for the choroid plexus epithelium (Supplementary Fig. S6). From these data, we conclude that choroid plexus epithelial cells express claudin-3 along with several other claudins.

Direct comparative qRT-PCR analysis of samples from freshly isolated choroid plexuses pooled from the lateral and 4^th^ ventricles of WT and claudin 3^−/−^ C57BL/6J mice confirmed expression of claudin-3 mRNA in the WT tissue (Fig. 6b). Interestingly, while expression levels for claudin-1, claudin-11 and occludin were comparable in choroid plexus samples from WT and claudin-3^−/−^ C57BL/6J mice, expression of claudin-2 mRNA was found to be significantly upregulated in choroid plexus samples isolated from claudin-3^−/−^ C57BL/6J mice compared to WT littermate samples. To determine if altered mRNA expression levels of claudin-2 translate to increased expression of claudin-2 protein in the choroid plexus of claudin-3^−/−^ C57BL/6J mice also at the protein level, we next performed side-by-side Western blot analysis on samples obtained from freshly isolated choroid plexuses pooled from the lateral and 4^th^ ventricles of WT and claudin 3^−/−^ C57BL/6J mice. We found enhanced expression of claudin-2 protein in choroid plexus samples from claudin 3^−/−^ C57BL/6J compared to WT C57BL/6J mice (Fig. 6c), while protein expression levels of claudin-11, β-catenin and occludin remained comparable (Fig. 6c and Supplementary Fig. S7). In addition to claudin-2, we also observed enhanced expression levels of claudin-1 protein in choroid plexus samples from claudin-3^−/−^ C57BL/6J mice when compared to that from WT mice, despite the identical mRNA levels (Fig. 6c). Taken together, lack of claudin-3 was accompanied by increased protein levels of claudin-2 and claudin-1 in the choroid plexus of C57BL/6J mice.

To finally determine if the concurrently altered expression levels of the cation and water channel-forming TJ protein claudin-2^20^ and of the barrier-forming claudin-1^21^ in the absence of claudin-3 affect BCSFB integrity *in vivo*, we performed *in vivo* permeability assays by investigating the diffusion of the exogenous tracers Evans blue, Hoechst dye and 3 kDa Dextran-TexasRed across the BCSFB. In brain tissue sections, Evans blue was readily detectable in the choroid plexus stroma of WT and claudin-3^−/−^ C57BL/6J mice, indicating unrestricted diffusion via the fenestrated choroid plexus microvessels (Fig. 6d). Similarly, Hoechst dye comparably stained the nuclei of the choroid plexus epithelial cells as visible in brain sections of both WT and claudin-3^−/−^ C57BL/6J mice (Fig. 6d). Diffusion of Evans blue in between the choroid plexus epithelial cells was visible to the same degree in the choroid plexus of WT and claudin-3^−/−^ C57BL/6J mice. There was also no diffusion of intravenously applied 3 kDa Dextran-TexasRed to the CSF of WT and claudin-3^−/−^ C57BL/6J mice (Fig. 6e), underscoring that absence of claudin-3 did not impair BCSFB integrity *in vivo*.

### Absence of claudin-3 does not affect the development of experimental autoimmune encephalomyelitis

**Figure 7:**
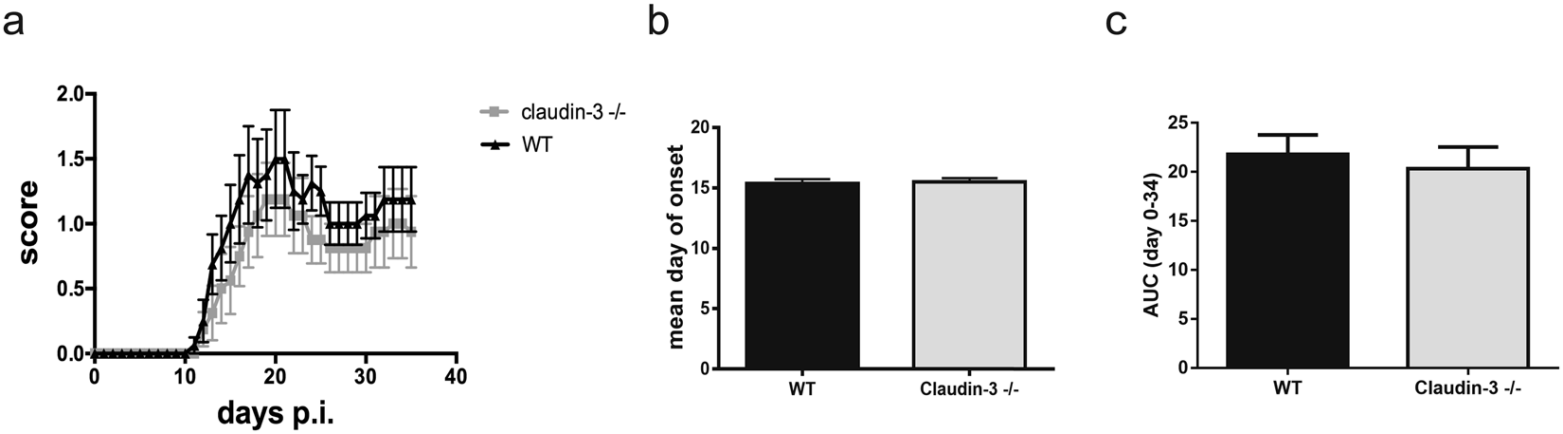
Absence of claudin-3 does not influence the development of active experimental autoimmune encephalomyelitis. (**a**) Graph of the clinical disease course of one representative MOG_aa35–55_ induced aEAE in WT (black line; n = 8) and claudin-3^−/−^ C57BL/6J mice (grey line; n = 8) is shown. Average disease scores ± SEM as assessed twice daily are shown. (**b**) Statistical analysis of the mean day of onset of disease between WT (n = 97) and claudin-3^−/−^ C57BL/6J (n = 89) mice from a total of ten different experiments. (**c**) Overall disease severity as determined by the area under the curve (AUC) and analyzed until day 35. Bar graphs represent the mean ± SD of four independent experiments including 45 WT and 45 claudin-3^−/−^ C57BL/6J mice.

Finally, we investigated if absence of claudin-3 in the BCSFB will affect the development of clinical EAE. To this end, EAE was induced in claudin-3^−/−^ C57BL/6J mice and WT littermates by immunization with MOG (myelin oligodendrocyte glycoprotein)_aa35–55_ peptide in complete Freund’s adjuvant (Fig. 7a). Investigating a total of 45 WT C57BL/6J mice and 45 claudin-3^−/−^ littermates in four individual EAE experiments, we did not detect any significant difference in the day of onset of clinical EAE (Fig. 7b), overall disease incidence (98% versus 96% in WT versus claudin-3^−/−^ C57BL/6J mice) and disease severity as determined by the area under the curve (Fig. 7c) in WT and claudin-3^−/−^ C57BL/6J mice. Thus, absence of claudin-3 does not aggravate EAE in C57BL/6J mice.

## Discussion

Breakdown of the endothelial BBB is a hallmark of neuroinflammatory disorders including MS and its animal model EAE^43,44^. Alterations of junctional complexes of the BBB as visualized by disrupted immunostainings for tight and adherens junction proteins, including JAM-A, JAM-B, occludin, ZO-1, claudin-5, claudin-3 and β-catenin have been shown to be intimately associated with BBB dysfunction in MS and EAE^23–25,45,46^. These studies, however, also revealed that it is notoriously difficult to reproducibly demonstrate junctional alterations at the BBB by means of immunostaining of tissue sections. Early reports showed immunostaining for claudin-1 at the BBB in mice^47^, which was found not to be expressed at the BBB^36^. Similarly, while we have reported immunostaining for claudin-3 in brain microvessels^31,45^, others could not confirm our observation^18,32^. These contradictory observations thus underscore the obvious difficulty in reliably detecting claudin localization in BBB TJs at the protein level.

In the present study, we therefore generated claudin-3^−/−^ C57BL/6J mice to investigate the function of claudin-3 in brain barrier TJs. Generation of claudin-deficient mice has proven a valuable tool allowing to understand the role of individual claudins in a given TJ complex^48^. This approach also provided evidence for the important role of claudin-5 in BBB TJs, where it regulates the diffusion of small molecules^8^. Previous identification of claudin-3 as a downstream target of the Wnt/β-catenin pathway^31^, which is critically involved in BBB maturation during development^49^, suggested potential involvement of claudin-3 in TJ maturation. Although our study does not directly address a role of claudin-3 in brain barrier maturation during embryonic development, our observation that claudin-3^−/−^ C57BL/6J mice were born at near mendelian ratios underscores that claudin-3 is not essential for brain barrier maturation during development.

Making use of our *in vitro* BBB model culturing pMBMECs from WT and claudin-3^−/−^ mice, we found that several anti-claudin-3 antibodies produced a junctional immunostaining pattern on both claudin-3^−/−^ and WT pMBMECs, which could not be accounted to cross-reactivity with claudin-5 or CLDND1. As this prohibited reliable detection of claudin-3 at the protein level we next investigated expression of claudin-3 in pMBMECs by RNAseq. While confirming our previous observations of high expression of claudin-5 and absence of claudin-1 in pMBMECs^36^, we did not also detect any mRNA for claudin-3 in the pMBMECs. To exclude that claudin-3 expression may be lost in cultured pMBMECs as observed for other junctional molecules before^50^, we confirmed lack of claudin-3 expression in brain endothelial cells *in vivo* by making use of our recent single cell RNA sequencing dataset^37^. In this dataset, we detected RNA sequences from a total of 21 different claudins. We found high expression of claudin-5 specifically in endothelial cells and confirmed previous reports on expression of claudin-12 in individual endothelial cells, as well as other cell lineages, and absence of claudin-1 from brain endothelial cells^8,36^. In contrast, claudin-3 was detected only in a total of 4 cells one of which clustered as endothelial cell. None of the other ≈1,500 endothelial cells analyzed showed claudin-3 expression. Claudin-3 thus belongs to the group of 13 claudins where RNA sequences were found in less than 10 cells and lacked cell specific assignment, which we interpreted as background of this experimental approach. Direct comparison of claudin-3 expression in freshly isolated brain microvessels from WT and claudin-3^−/−^ C57BL/6J mice by qRT-PCR confirmed the unexpected absence of claudin-3 mRNA in brain endothelial cells *ex vivo*. Thus, the availability of claudin-3^−/−^ C57BL/6J mice has allowed to provide compelling evidence that mouse brain microvascular endothelial cells do not express claudin-3 mRNA.

Availability of claudin-3^−/−^ C57BL/6 mice allowed us to demonstrate that some but not all anti-claudin3 antibodies produced junctional immunostaining in brain microvessels also in tissue sections from claudin-3^−/−^ C57BL/6J mice underscoring that these anti-claudin-3 antibodies cross-react with an unknown endothelial junctional component present in claudin-3^−/−^ mice.

Our study confirms expression of claudin-3 mRNA and protein and its specific detection in choroid plexus epithelial cells forming the BCSFB *in vivo* and *in vitro,* as previously observed by others^17,18,39,51^. All anti-claudin-3 antibodies that reliably produced a junctional immunostaining of the BCSFB *in vivo* and *in vitro* of WT mice did not produce any immunostaining on the BCSFB of claudin-3^−/−^ C57BL/6J mice. Unfortunately, our monoclonal antibodies targeting the extracellular domain of claudin-3 failed to detect endogenous claudin-3 in the BCSFB *in situ* and *in vitro*. This may be due to differences in the posttranslational modifications of endogenous claudin-3 versus claudin-3 expressed in heterologous cells in claudin-3^−/−^ mice following vaccination with the claudin-3 expression vectors, or to lack of accessibility of the extracellular domains of claudin-3 within the BCSFB TJs.

Expression of claudin-3 at the BCSFB *in vivo* could be confirmed with our first single cell RNAseq data sets with mixed transcriptomes underscoring that our early applied brain endothelial or brain pericyte purification protocols failed to produce the highly purified cellular subsets. Highly specific expression of claudin-3 was confirmed in a cluster of sorted cells identified as choroid plexus epithelial cells due to expression of signature genes such as Ttr and the cytokeratins Krt8, Krt18 and Krt23. The latter observations also highlight that potential contaminations of the brain microvascular endothelial cell preparations need to be considered when aiming to study their gene expression profile. While single cell RNAseq followed by cluster analysis will display heterogeneity of purified brain cellular subsets, mRNA expression studies of isolated brain microvessels by microarray or bulk RNAseq analysis will assign detection of these mRNAs to brain endothelium unless carefully tested for potential contaminants^14^. Considered contamination with pericytes or astrocytes^14,52^, most studies underestimate potential contamination with choroid plexus epithelial cells due to co-isolation of choroid plexus stromal microvascular endothelial cells from these highly vascularized structures^53^. In fact, several studies including ours have detected low expression of claudin-3 mRNA in freshly isolated brain microvessels^15,52,54^, which may thus rather be due to contaminations with choroid plexus epithelium.

Direct comparison of claudin-3 expression in freshly isolated choroid plexus from WT and claudin-3^−/−^ C57BL/6J mice by qRT-PCR underscored expression of claudin-3 mRNA in the choroid plexus *in vivo*. In both samples, detection of claudin-1, claudin-2 and claudin-11 confirmed previous observations^17^. Interestingly, in the absence of claudin-3, expression of the pore forming claudin-2 at the mRNA and protein level was upregulated, which may lead to increased leakiness of the BCSFB in claudin3^−/−^ mice as previously observed^39^. However, at the same time we detected upregulated protein expression of the TJ sealing claudin-1^21^, which may compensate the TJs weakening role of claudin-2 in the absence of claudin-3. Absence of claudin-3 in BCSFB TJs may thus lead to compensatory stabilization of claudin-1 and claudin-2 to maintain overall TJs architecture and function. Indeed, *in vivo* tracer diffusion studies confirmed BCSFB integrity in healthy claudin-3^−/−^ C57BL/6J mice. Finally, in apparent contrast to a previous study^39^ we also did not detect any impaired BCSFB function in claudin-3^−/−^ C57BL/6J mice during EAE leading to aggravation of the clinical disease when compared to WT C57BL/6J mice. This previous study employed different strategies for targeted deletion of claudin-3 and subsequent breeding of mice with two different C57BL/6 strains, namely C57BL/6 N and C57BL/6 C. As these two C57BL/6 substrains display significant behavioral differences^55^, the heterogeneous genetic background of the individual animals compared in this prior investigation could also impact on EAE pathogenesis. Our present study excludes such genetic variations on EAE pathogenesis as it compared disease course in claudin-3^−/−^ C57BL/6J mice backcrossed at least 10 times to this genetic background with WT littermates.

Taken together, establishing claudin-3^−/−^ C57BL/6J mice has allowed to demonstrate that the junctional immunostaining produced by anti-claudin-3 antibodies in mouse brain endothelial cells *in situ* and *in vitro* is not due to claudin-3 presence but rather to an endothelial junctional antigen that is still present in brain endothelial cells of claudin-3^−/−^ mice. Detection of claudin-3 mRNA in brain microvascular preparations thus needs to be reconsidered as potential contamination of these preparations with choroid plexus epithelium. While the present study did not reveal a role for claudin-3 in the BCSFB in mice, a potential role for claudin-3 in regulating BCSFB function in man remains to be investigated.

## Material and Methods

### Mouse housing

Mice were housed in individually ventilated cages under specific pathogen-free conditions at 22 °C with free access to chow and water. Animal procedures executed were either approved by the Veterinary Office of the Canton Bern (permit no. BE42/14) or by the Uppsala Ethical Committee on Animal Research (Permit number: C224/12 and C115/15) and the Stockholm North Animal Ethics committee (Stockholms Norra Djurförsöksetiska Nämnd), permit N150/14 and are in keeping with institutional and standard protocols for the care and use of laboratory animals in Switzerland and Sweden.

### Generation of claudin-3 deficient mice

Four overlapping clones encoding the mouse claudin-3 gene were obtained by screening a λ 129/Sv genomic library. Using two of them, the targeting vector was constructed by ligating a 5.0-kb fragment located upstream of the Not I site of the only exon and a 2.0-kb fragment located downstream of the Kpn I site of the exon as the 5′ and 3′ arm, respectively, to the PGK-neo cassette. The diphtheria toxin A expression cassette (MC1pDT-A) was placed outside the 3′ arm for negative selection. This targeting vector was designed to delete most of the coding region of claudin-3 except a part encoding the C-terminal 10 amino acids. J1 ES cells were electroporated with the targeting vector and selected for ~9 days in the presence of G418. The G418-resistant colonies were collected, expanded and screened by Southern blotting with the 3′ external probe. Correctly targeted ES clones were identified by an additional 6.4-kb band together with the 17.7 kb band of the WT allele, when digested with BamHI. The targeted ES cells obtained were injected into C57BL/6J blastocysts, which were in turn transferred into BALB/c foster mothers to obtain chimeric mice. Male chimeras were mated with C57BL/6J females, and agouti offspring were genotyped to confirm germline transmission of the targeted allele. Heterozygous mice were intercrossed with WT C57BL/6J mice (Janvier-Labs; Saint-Berthevin Cedex, France) for at least 10 generation. Offspring were routinely genotyped by standard genomic PCR using the following primers RVP GT-1 (5′-AAGATCACCATCGTGGCGGGA-3′), RVP GT-2 (5′- TCAGACGTAGTCCTTGCGGTC-3′) and Loxneo5′ (5′-CATGCTCCAGACTGCCTT-3′) at an annealing temperature of 55 °C giving rise to a 200 bp PCR product from the claudin-3 mutant allele and a 317 bp product from the WT allele. Finally, heterozygous mice were interbred to obtain homozygous mice and WT littermates to be used in experimental cohorts.

### Southern Blotting

Genomic DNA was isolated from snap-frozen spleens of adult mice previously PCR genotyped as WT, heterozygote or claudin-3^−/−^. Briefly, spleens were ground to a fine powder in a mortar filled with liquid nitrogen and then transferred to a proteinase-K containing digestion buffer and incubated overnight at 50 °C. For purification, the solutions were extracted with equal volumes of phenol/chloroform/isoamyl (25:24:1). After centrifugation, the aqueous layers were transferred to new tubes and 0.5 volumes of 7.5 M ammonium acetate and 2 volumes of 100% ethanol were added and mixed by inversion. After centrifugation, the pellet was rinsed with 70% ethanol and then air-dried. The DNA was dissolved in Tris-EDTA buffer. To increase the purity of the DNA, dialysis was performed against TE buffer at 4 °C and it was validated by taking OD readings at 260 and 280 nm. Genomic DNA was digested with BamHI and separated by agarose gel-electrophoresis. DNA fragments were transferred to a Hybond N +membrane by capillary transfer and incubated with digoxigenin-labeled external probe fragment.

### Experimental autoimmune encephalomyelitis

EAE was induced in 8–12 week-old female C57BL/6J claudin-3^−/−^ mice and their C57BL/6J WT littermates and scored exactly as described^56–58^.

### *In vitro* brain barrier models and cell lines

pMBMECs and pMCPECs were isolated from 7–9 week old claudin-3^−/−^ C57BL/6J mice and their WT littermates and cultured and tested for their barrier characteristics exactly as described^33,40,59,60^. L-claudin-1 and L-claudin-3 fibroblast cell lines were established as previously described^61^. HEK-293-claudin-5-YFP cell line was kindly provided by Ingolf Blasig^62^.

### Assessment of brain barrier integrity *in vitro*

*In vitro* permeability of pMBMEC and pMCPEC monolayers to AlexaFluor 680-labelled 3 kDa-dextran (Thermo Fisher Scientific, Carlsbad, CA, USA) and to Lucifer Yellow (LY; 457 Da) (Sigma-Aldrich Chemie GmbH, Buchs, Switzerland) was assessed exactly as described^40,60^. TEER of monolayers formed by pMCPECs and pMBMECs, respectively, was assessed by impedance TEER measurements using the cellZscope device, as described previously^40^.

### RNA sequencing

RNA was extracted from pMBMECs with the High Pure RNA Isolation Kit (Hoffman-La Roche, Basel, Switzerland). RNA libraries for 5 replicates were prepared according to the manufacturer’s instruction using TruSeq stranded mRNA Library Prep Kit with polyA selection. The quality of the samples was assessed using a Fragment analyser with the samples having RIN values ≥8 and an average of 9.2.

For the analysis, generated fastq files were checked for quality measures using FASTQC software (version 0.11.2). Depth of sequencing was in average ~25 M reads for each of the 15 replicates. The few rRNA fragments that were still present despite the polyA selection were removed using Trimmomatic (version 0.33)^63^ followed by an additional round of quality control. The fastq files were then aligned to the mm10 reference genome using TopHat2 (version 2.0.13)^64^ after which Qualimap (version 2.2)^65^ was used for quality control and IGV (version 2.3.69)^66^ for visualization of the aligned reads. Counts by transcript were evaluated using HTSeq-count (version0.6.1)^67^ and normalization was performed with the DESeq. 2 package (Version 1.12.4)^68^ in R (Version 3.2.2).

### Single cell RNA sequencing

Single cell RNA sequencing was performed and the data analyzed as described^37^. The data analyzed herein are partly taken from the pusblished dataset^37,38^ and available at http://betsholtzlab.org/VascularSingleCells/database.html. However, part of the data are also from an unpublished dataset, in which choroid plexus epithelial cells were obtained as contaminants when sorting for endothelial cells and pericytes. For this dataset, a slightly different protocol was used for single cell dissociation, leading to a high proportion of incompletely dissociated cells. Hence, the choroid plexus epithelial cells analyzed contain fragements of associated endothelial cells and/or pericytes.

### Quantitative Real-Time PCR Analysis (qRT-PCR)

After RNA extraction from freshly isolated mouse choroid plexus and brain microvessels and from pMBMEC and pMCPEC cultures, cDNA was obtained from each sample’s total isolated RNA with the SuperScript III First-Strand Synthesis System (Invitrogen, Carlsbad, CA, USA) and the qRT-PCR was done as previously described^52^. The sequences of the primers used for each gene are presented in Supplementary Table S4 and the average of C_T_ values of the analyzed genes in this study is presented in Supplementary Table S3.

### SDS-PAGE

Liver tissue, pMBMECs, freshly isolated microvessels and the choroid plexus were lysed in HES lysis buffer (10 mM Hepes, 1 mM EDTA solution, 250 mM sucrose solution), with protease inhibitors. Samples were separated by electrophoresis in a 10% SDS-polyacrylamide gel and transferred to a nitrocellulose membrane. Membranes were blocked with Rockland Buffer for 1 hour, RT and incubated with the respective primary antibodies overnight at 4 °C (Supplementary Table S5). After the washing step, membranes were incubated with the secondary antibodies for 1 hour at RT (Supplementary Table S5). Proteins were detected using an Odyssey Infrared Imaging system. Band intensity was quantified using the ImageJ software and normalized against β-actin.

### Immunofluorescence staining *in vitro*

Confluent cell lines, pMBMEC and pMCPEC monolayers were stained exactly as described^40^. Primary and secondary antibodies are described in Supplementary Table S5. Fluorescence stainings were analyzed using a Nikon Eclipse E600 microscope connected to a Nikon Digital Camera DXM1200F with the Nikon NIS-Elements BR3.10 software (Nikon, Egg, Switzerland). Images were processed and mounted using Adobe Illustrator software.

### Immunofluorescence staining of tissue sections

Mice were anesthetized with Isoflurane Baxter (Arovet, Dietikon, Switzerland) and perfused with 1% PFA. Livers and brains were removed, embedded in Tissue-Tek O.C.T. compound (Sakura Finetek, The Netherlands) and snap-frozen. Cryosections were cut at 6 μm thickness, and either fixed in ice cold acetone for 10 min or fixed for 10 min in 100% EtOH, 4 °C, and 1 min in acetone, RT, depending on the used primary antibody. Cryosections were stained as described before^69,70^. Primary and secondary antibodies are described in Supplementary Table S5. Fluorescence stainings were analyzed using a Nikon Eclipse E600 microscope connected to a Nikon Digital Camera DXM1200F with the Nikon NIS-Elements BR3.10 software. Images were processed and mounted using Adobe Illustrator software.

### *In vivo* permeability assay

Mice received an intravenous injection of 2% Hoechst 33258 and 2% Evans Blue in 100 µL PBS at a ratio of 20:80 (v/v). Dyes circulated for 30 min and 15 min, respectively. Brains from Hoechst/Evans Blue-injected mice were immediately snap-frozen in Tissue-Tek O.C.T. compound. Cryosections (6 µm) were analyzed for dye extravasation from brain, with extravasation across the fenestrated endothelium of the choroid plexus serving as positive control.

### Cerebrospinal fluid extraction

Mice received 1 mL NaCl subcutaneous 30 minutes before the beginning of the assay. 5 mg/mL of 3 kDa Dextran-TexasRed were then administrated by tail vein injection and were in circulation for 15 minutes. Afterwards, mice were anesthetized with 100 µL of FeMiMe (0.05 mg/mL Fentany, 15 mg/3 mL Dormicum, 1 mg/mL Dormitor), intramuscular, and cisterna magna was assessed as previously described^71^. From each mouse, CSF was harvested with a glass capillary. Total volume of CSF from all the mice was pooled together and diluted 1:1 in NaCl, to reach a final volume of 50 µL, required for the absorbance measurement. Blood was collected in heparin-tubes and plasma was obtained from it after centrifugation for 10 minutes, 4 °C, 4000 RPM. Equal volumes of plasma and CSF were loaded in a 96-well microplate and absorbance was measured.

### Statistics

Statistical analysis was performed using GraphPad Prism 6.0 software. To compare two groups, an unpaired t-test with Welch’s correction was performed and for the comparison of three groups, we performed a one-way ANOVA with post-hoc Tukey test. For the assessment of brain barrier integrity *in vitro* and for the analysis of the EAE, a Mann-Whitney U-test was performed. Results are shown as mean ± SD and a p < 0.05 was considered significant.

## Electronic supplementary material

Supplementary Information
