# Publisher Correction: Claudin-3-deficient C57BL/6J mice display intact brain barriers

**DOI:** 10.1038/s41598-019-43511-0

**Published:** 2019-07-18

**Authors:** Mariana Castro Dias, Caroline Coisne, Ivana Lazarevic, Pascale Baden, Masaki Hata, Noriko Iwamoto, David Miguel Ferreira Francisco, Michael Vanlandewijck, Liqun He, Felix A. Baier, Deborah Stroka, Rémy Bruggmann, Ruth Lyck, Gaby Enzmann, Urban Deutsch, Christer Betsholtz, Mikio Furuse, Shoichiro Tsukita, Britta Engelhardt

**Affiliations:** 1 Theodor Kocher Institute, University of Bern, Bern, Switzerland; 2 Laboratory of Tumor Immunology and Cell Therapy, Hyogo College of Medicine, Nishinomiya, Hyogo Japan; 3 Division of Cell Biology, Kobe University Graduate School of Medicine, Kobe, Hyogo Japan; 4 Interfaculty Bioinformatics Unit and Swiss Institute of Bioinformatics, University of Bern, Bern, Switzerland; 5 Karolinska Institutet/AstraZeneca Integrated Cardio Metabolic Centre (KI/AZ ICMC), Huddinge, Sweden; 6 Visceral Surgery Research Laboratory, Department of Biomedical Research, University of Bern, Bern, Switzerland; 7 Department of Immunology, Genetics and Pathology, Rudbeck Laboratory, Uppsala University, Uppsala, Sweden; 8 Division of Cell Structure, National Institute for Physiological Sciences, Okazaki, Japan; 9 Department of Physiological Sciences, School of Life Science, Okazaki, Japan; 10 Department of Cell Biology, Kyoto University Faculty of Medicine, Kyoto, Japan

Correction to: *Scientific Reports* 10.1038/s41598-018-36731-3, published online 18 January 2019

In the original version of this Article, Shoichiro Tsukita (deceased) was incorrectly affiliated with ‘Theodor Kocher Institute, University of Bern, Bern, Switzerland’. The correct affiliation for Shoichiro Tsukita is listed below:

Department of Cell Biology, Kyoto University Faculty of Medicine, Kyoto, Japan.

This error has now been corrected in the PDF and HTML versions of the Article, and in the accompanying Supplementary Material.

