# Supplementary material for: Claudin-3-deficient C57BL/6J mice display intact brain barriers
Source: Sci Rep. 2019 Jan 18;9:203. doi: 10.1038/s41598-018-36731-3 (PMC6338742; doi:10.1038/s41598-018-36731-3)
Supplement: Supplementary file 1 — Supplementary Information [file 41598_2018_36731_MOESM1_ESM.pdf]

Supplementary Information

Claudin-3-deficient C57BL/6J mice display intact brain barriers

Mariana Castro Dias[1,$], Caroline Coisne[1,$], Ivana Lazarevic[1], Pascale Baden[1], Masaki Hata[2], Noriko Iwamoto[3], David Miguel Ferreira Francisco[4], Michael Vanlandewijck[5], Liqun He[5], Felix A. Baier[6], Deborah Stroka[6], Rémy Bruggmann[4], Ruth Lyck[1], Gaby Enzmann[1], Urban Deutsch[1], Christer Betsholtz [5,7], Mikio Furuse[8,9], Shoichiro Tsukita[10] and Britta Engelhardt[1*]

[1] Theodor Kocher Institute, University of Bern, Switzerland; [2]Laboratory of Tumor Immunology and Cell Therapy, Hyogo College of Medicine, Japan; [3]Division of Cell Biology, Kobe University Graduate School of Medicine, Japan; [4]Interfaculty Bioinformatics Unit and Swiss Institute of Bioinformatics, University of Bern, Switzerland; [5]Karolinska Institutet/ AstraZeneca Integrated Cardio Metabolic Centre (KI/AZ ICMC), Huddinge, Sweden; [6] Visceral Surgery Research Laboratory, Department of Biomedical Research, University of Bern, Switzerland; [7] Department of Immunology, Genetics and Pathology, Rudbeck Laboratory, Uppsala University, Sweden; [8]Division of Cell Structure, National Institute for Physiological Sciences, Okazaki, Japan; [9]Department of Physiological Sciences, School of Life Science, Okazaki, Japan; [10]Deceased, Department of Cell Biology, Kyoto University Faculty of Medicine, Japan.

[$] these authors contributed equally to this work

This project has been funded by the European Union (EU) Seventh Framework Program FP7 under grant agreements 241861 (JUSTBRAIN) and 607962 (nEUROinflammation) and the EU Horizon 2020 MSCA-ITN-2015 675619 BtRAIN.

***Corresponding author:**
Prof. Dr. Britta Engelhardt
Theodor Kocher Institute
University of Bern
Freiestr. 1
CH-3012 Bern
Switzerland
Tel. + 41 31 631 4143
E-Mail: bengel@tki.unibe.ch

Supplementary Figure S1

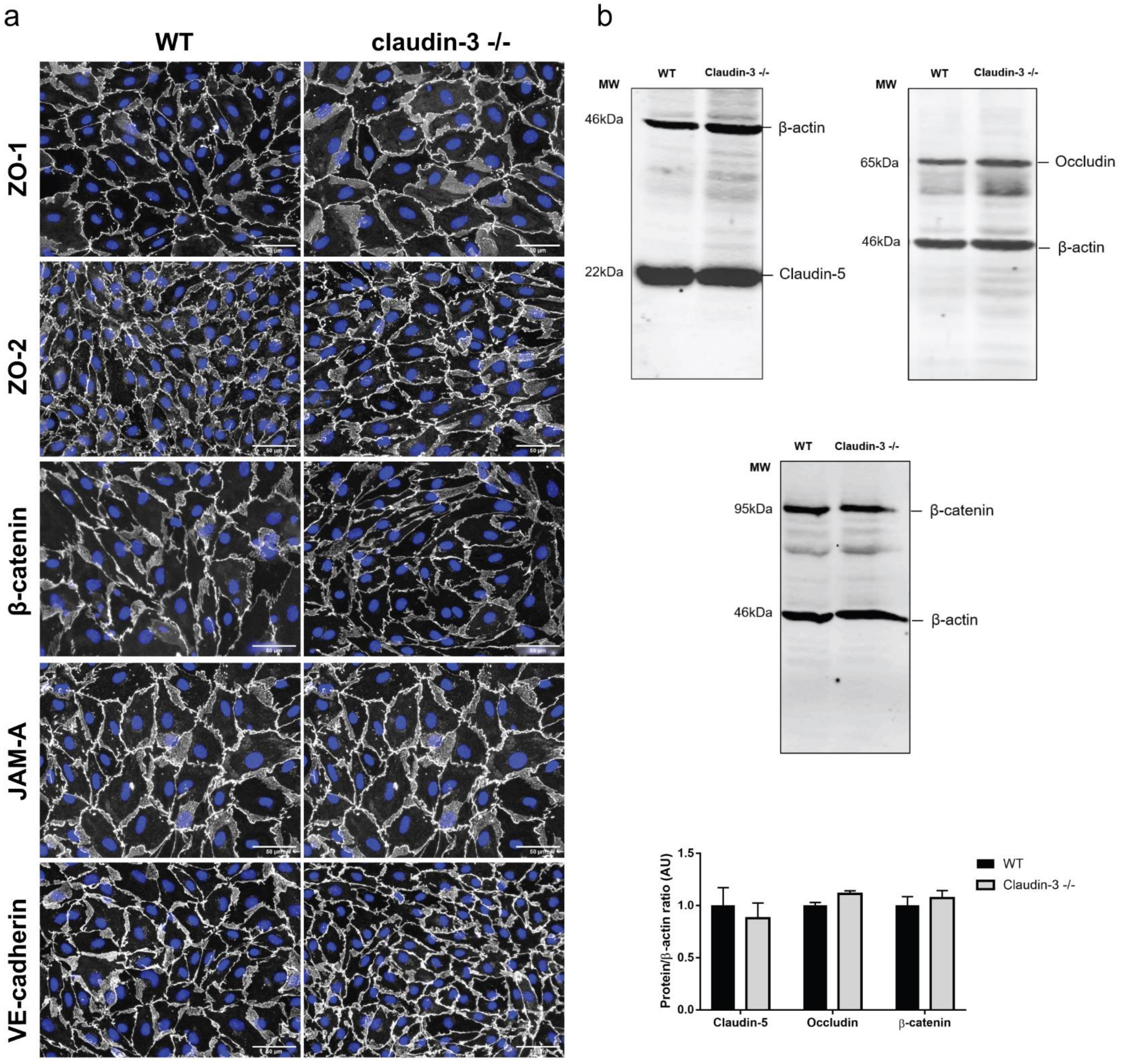

Supplementary Figure S1. (a) Comparable junctional immunofluorescence staining for ZO-1, ZO-2, β-catenin, JAM-A and VE-cadherin is detected in WT and claudin-3-/- pMBMEC monolayers. Scale bar=50µm. **(b)** Immunoblot analysis for claudin-5, occludin and β-catenin in samples of freshly isolated pMBMECs pooled from 4 WT and 4 claudin-3-/- C57BL/6J mice. For each protein, three independent pMBMEC samples from WT and claudin-3-/- C57BL/6J mice were analysed. Bar graph shows mean +/- SD of three independent experiments.

Supplementary Figure S2

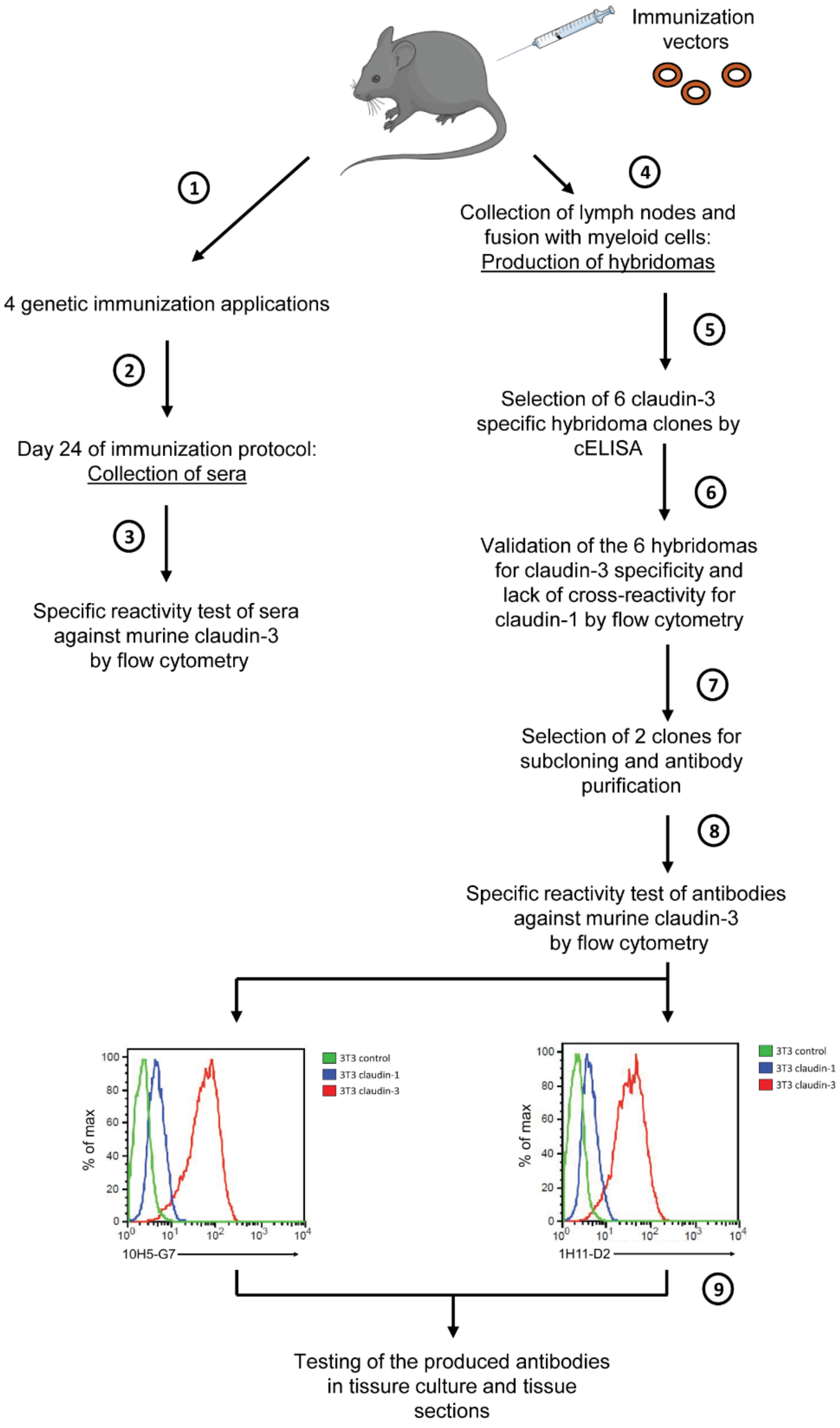

Supplementary Figure S2: Schematic representation of the production of the monoclonal mouse anti-mouse claudin-3 antibodies. Flow cytometry analysis of claudin-3 expression in L929 cells transfected with claudin-1 and claudin-3 is shown, using the anti-claudin-3 clones 10H5-G7 and 1H11-D2. Both antibodies were used at a concentration of 10 µg/mL and non-transfected L929 cells were used as negative control. Histogram plots show cells scatter gated on live cells. Samples were measured on BD FACSCalibur using CellQuest software. Post-acquisition analysis was performed using the FlowJo (ver 10) software. Mouse and syringe cartoon were adapted from Servier Medical Art (http://smart.servier.com/), licensed under a Creative Common Attribution 3.0 Generic License.

Supplementary Figure S2 extended legend

Production of monoclonal antibodies recognizing claudin-3 extracellular domains

Generation of hybridomas producing monoclonal antibodies against claudin-3 extracellular domains was performed by Aldevron (Freiburg, Germany; Supplementary Figure 1). To this end, a codon optimized cDNA of the “long” splice variant of mouse claudin-3 was cloned into an Aldevron proprietary immunization vector (pSCF9). pSCF9-claudin-3-mur-FL was transiently transfected into mammalian cells. Cell surface expression of the resulting protein containing a vector-derived N-terminal *tag*-sequence, was analyzed by flow cytometry on non-fixed, living cells. Cell surface expression of pSCF9-claudin-3- mur-FL (long variant) was demonstrated.

Five claudin-3-/- C57BL/6J mice were immunized with a mix of the immunization vectors pSCF9-claudin- 3-mur-FL and pCMV-Sport6-claudin-3-mur-FL-long-fl-long. The immune serum was taken at day 24 of the immunization protocol, after 4 genetic immunization applications. Sera were tested by Aldevron by flow cytometry using mammalian cells transiently transfected with the mouse claudin-3 cDNA (claudin- 3-mur) cloned into an Aldevron proprietary expression vector (pSCF9-claudin-3-mur-FL-long) and pCMV-Sport6-claudin-3-mur-FL-long. Specific reactivity of the immune sera against cells transfected with pSCF9-claudin-3-mur-FL-long and pCMV-Sport6-claudin-3-mur-FL-long, respectively, could be detected in the sera from all immunized claudin-3-/- mice when compared to cells transfected with an irrelevant cDNA. Sera from the immunized mice were additionally tested in our laboratory by flow cytometry of 3T3 cells stably expressing mouse claudin-1 or claudin-3. All sera stained claudin-3 expressing cells, while sera from select mice cross-reacted with claudin-1 expressing cells. At Aldevron, lymph node cells were collected from all 5 immunized mice, which were then fused with myeloma cells and hybridomas were established and cloned by limited dilution for monoclonal antibody production. Hybridoma supernatants were screened by cell-based ELISA using transiently transfected cells leading finally to the selection of 6 clones, which were further characterized for claudin-3 detection and lack of claudin-1 and claudin-5 cross-reactivity by flow cytometry. 3 of the 6 hybridoma clones were subcloned. Supernatants and purified antibodies from these 3 clones were produced and re-tested for claudin-3 detection in transfectants and lack of cross-reactivity for claudin-1 and claudin-5. Fulfilling these selection criteria these antibodies were used for immunostainings in tissue sections and on cultured cells. Schematic representation of the full procedure is illustrated in Supplementary Figure S2.

Supplementary Figure S3

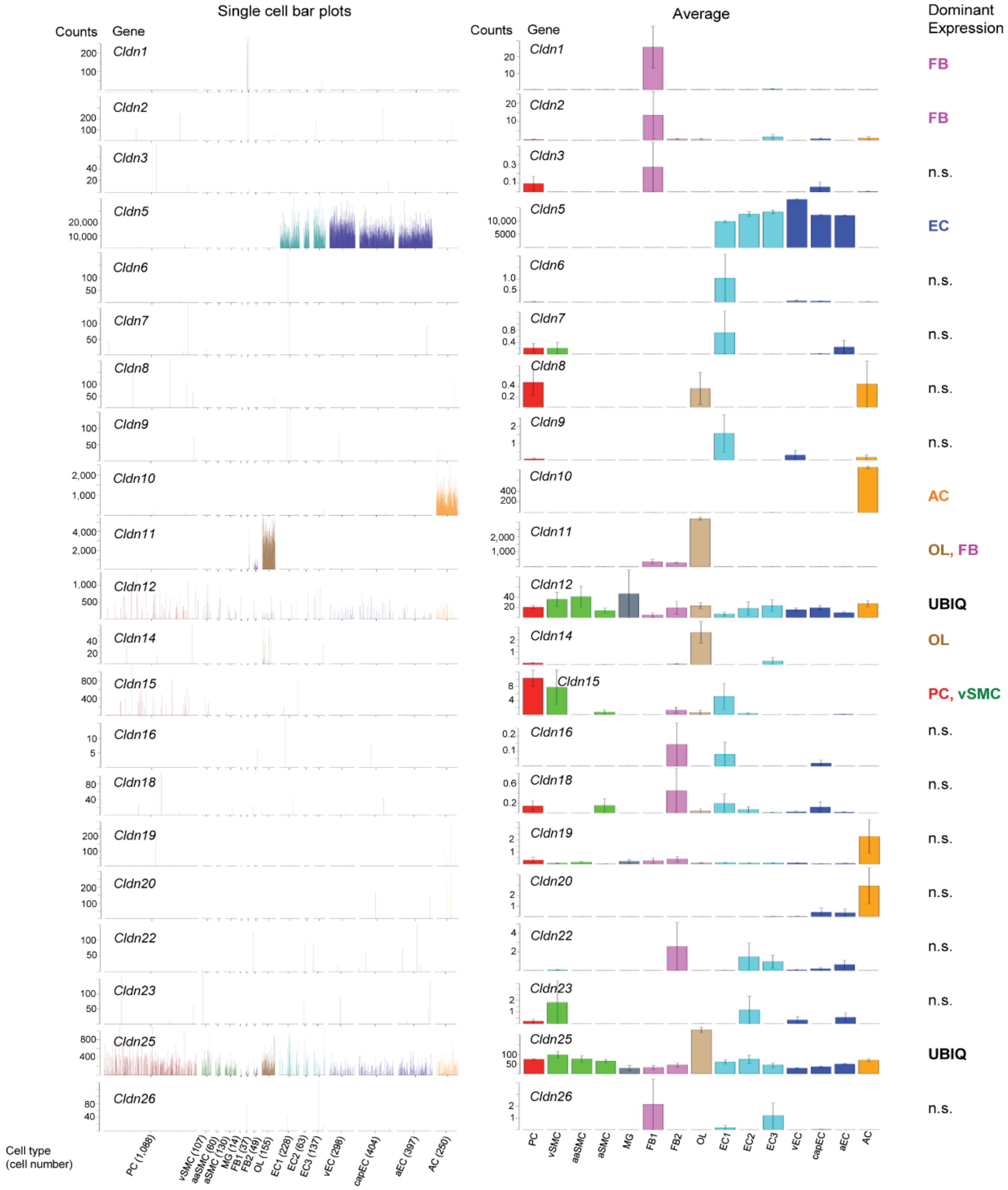

PC = pericyte
SMC = smooth muscle cell
MG = microglia
FB = perivascular/meningeal fibroblast-like cell

OL = oligodendrocyte lineage cell
EC = endothelial cell
AC = astrocytes
1,2,3 = subtype
n.s. = not significant

UBIQ = ubiquitous
v = venous
aa = arteriolar
a = arterial
cap = capillary

Supplementary Figure S3: Expression of claudins (Cldn) at the BBB depicted by single cell RNA sequencing. The graphs shown are excerpts from http://betsholtzlab.org/VascularSingleCells/database.html [37 38] and represent the expression of all claudins for which RNA sequences were detected in at least one cell. The left panel of graphs represents bar plots where each cell has unique position along the x-axis. Cell type abbreviations and numbers, within brackets, are provided at the bottom. The right panel of graphs shows average values and standard deviation. Note the huge difference in expression counts on the y-axes. For most of the claudins, including claudin-3, sequences were recorded in only a few cells and without cell type bias; for these, the dominant expression (rightmost part of the figure) was deemed not significant. However, for 9 of the claudins, a dominant expression pattern was noted as indicated.

Supplementary Figure S4

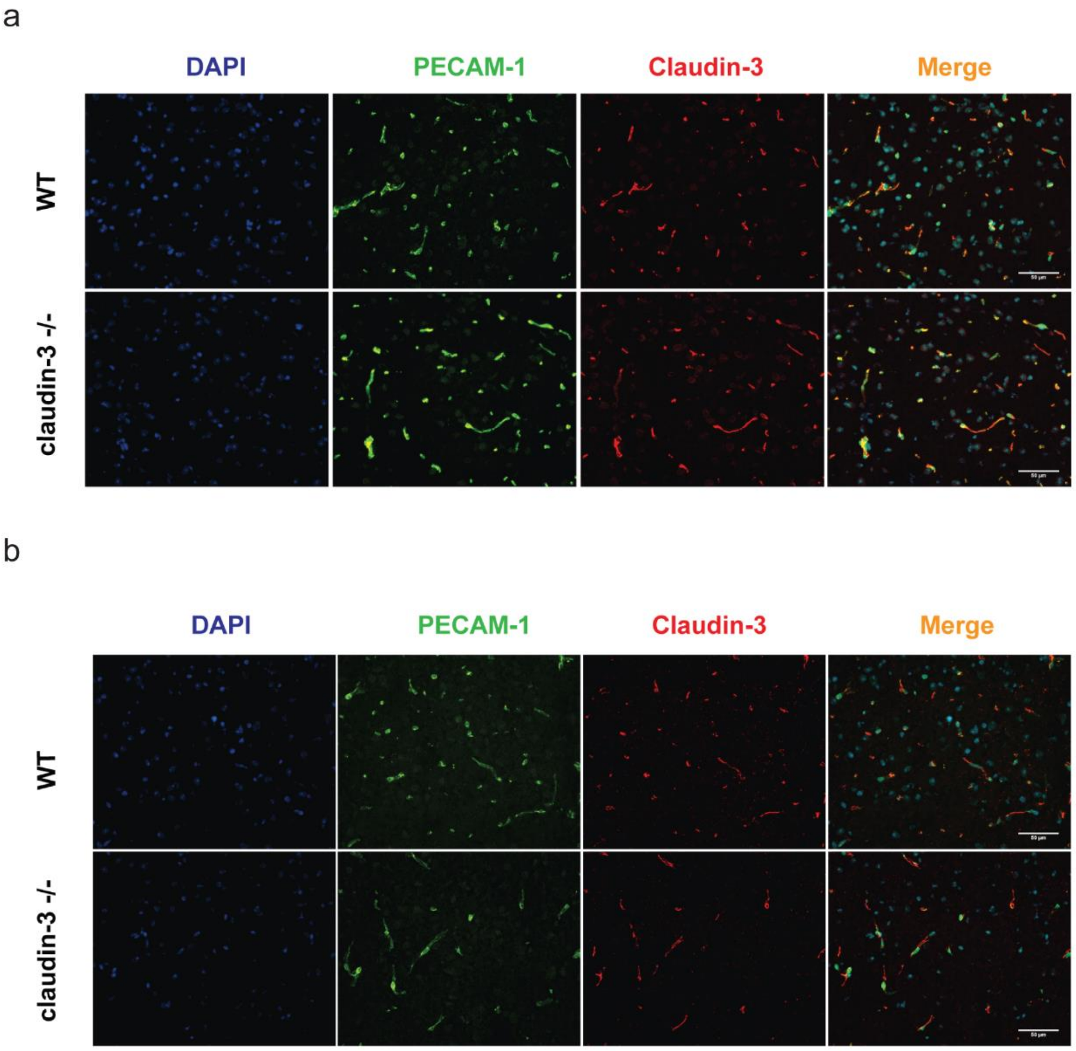

Supplementary Figure S4: Immunofluorescence staining of frozen brain sections from WT and claudin-3-/- C57BL/6J mice for PECAM-1 (green) and claudin-3 (red). Nuclei are stained with DAPI (blue). **(a)** Anti-claudin-3 antibody from Invitrogen and **(b)** anti-claudin-3 antibody from Abnova stain brain microvessels in WT and claudin-3-/- C57BL/6J mice, which confirms false positive detection of claudin-3 at the BBB due to cross-reactivity with an unknown endothelial antigen. Two independent stainings were performed. Scale bars = 50 µm.

Supplementary Figure S5

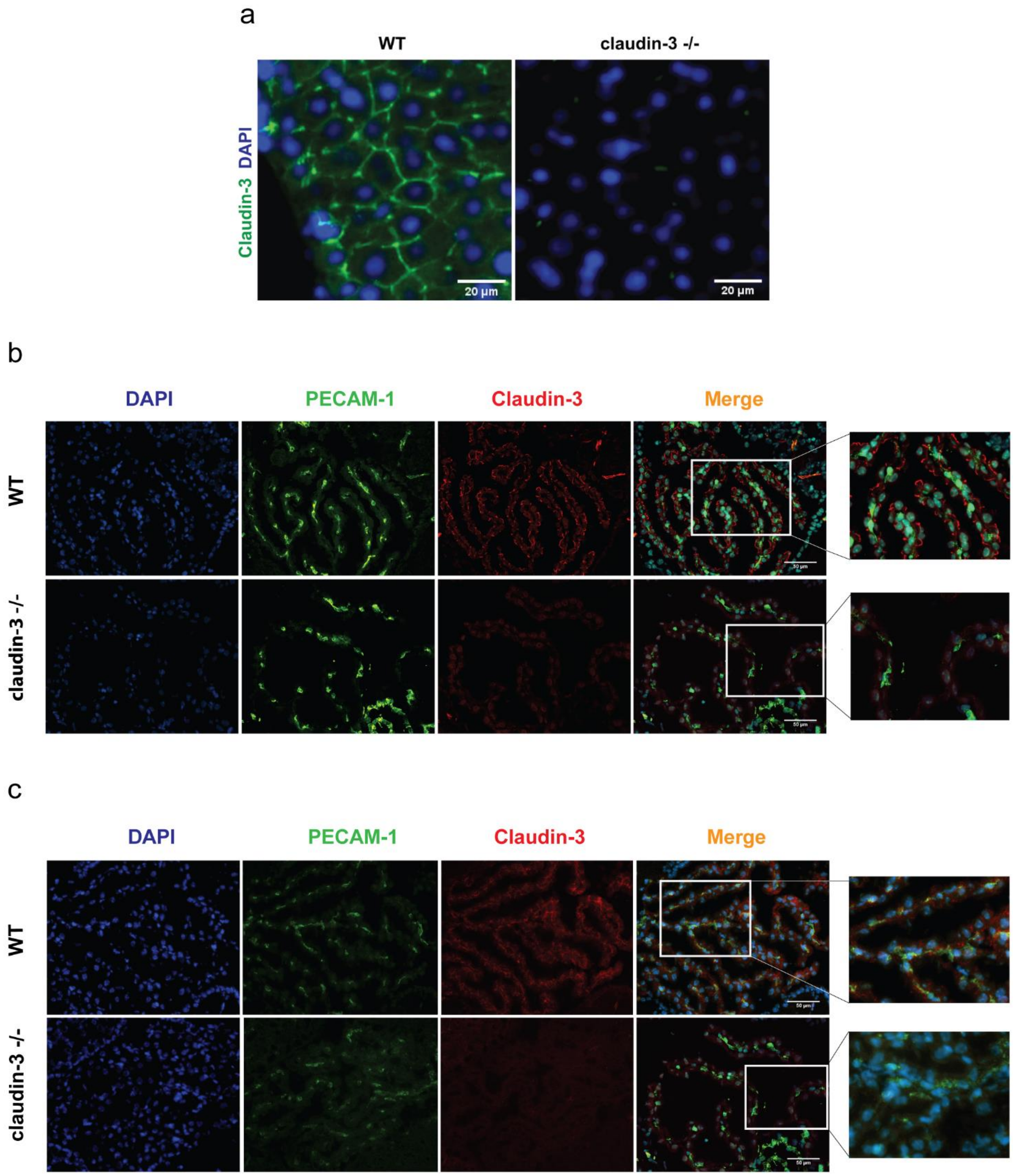

Supplementary Figure S5: Immunofluorescence staining of frozen liver sections **(a)** and of brain sections **(b and c)** from WT and claudin-3-/- C57BL/6J mice. A polyclonal anti-claudin-3 antibody (Novus Biologicals) **(a and c)** and polyclonal anti-claudin-3 antibody (Invitrogen) **(b)** stain cellular junctions in liver and epithelial junctions in the choroid plexus of WT but not of claudin-3-/- C57BL/6J mice. In the brain sections, note the lack of overlap of claudin-3 staining with PECAM-1 immunostaining in the merge which is even more clearly visible in the blow ups from the boxed areas shown to the right. Three independent stainings were done. Scale bars=20 µm in **(a),** 50 µm in **(b and c).**

Supplementary Figure S6

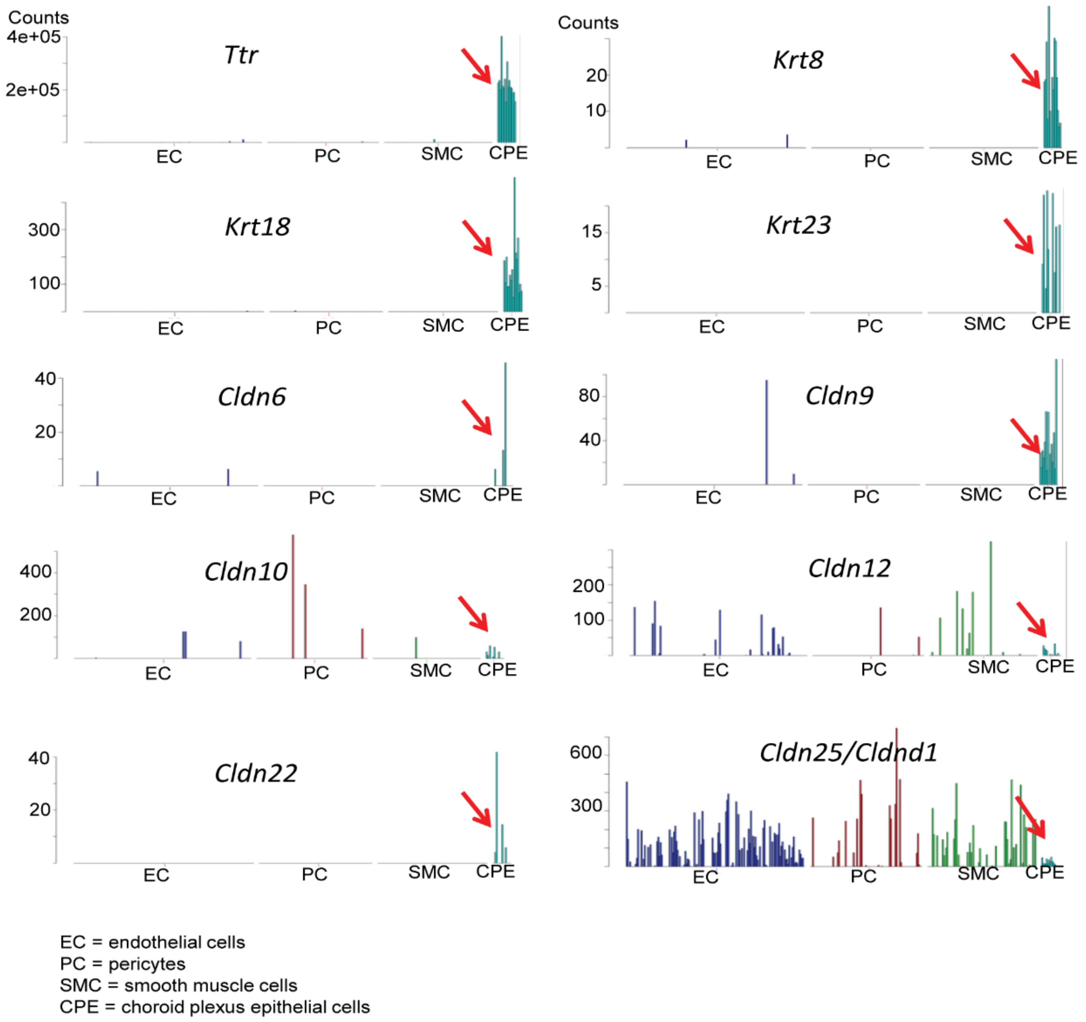

Supplementary Figure S6: Expression of claudins in choroid plexus epithelial cells depicted by single cell RNA sequencing. The graphs are from a dataset distinct from that available at http://betsholtzlab.org/VascularSingleCells/database.html [37 38]. In this dataset a small but distinct set of choroid plexus epithelial cells (CPE, indicated by red arrows) were identified based on the highly specific marker Ttr; note the order of magnitude of expression of this gene relative to all other genes. The identity of the CPE cluster is further supported by the expression of the highly specific marker Ttr and of several cytokeratins, including Krt8, Krt18 and Krt23. Additional claudins displaying significant expression in the CPE cluster are shown. These include claudin-6, -9, -10, -12, -22 and -25/CLDND1. Note the differences in sequence counts (Y-axes) reflecting different levels of RNA expression.

Supplementary Figure S7

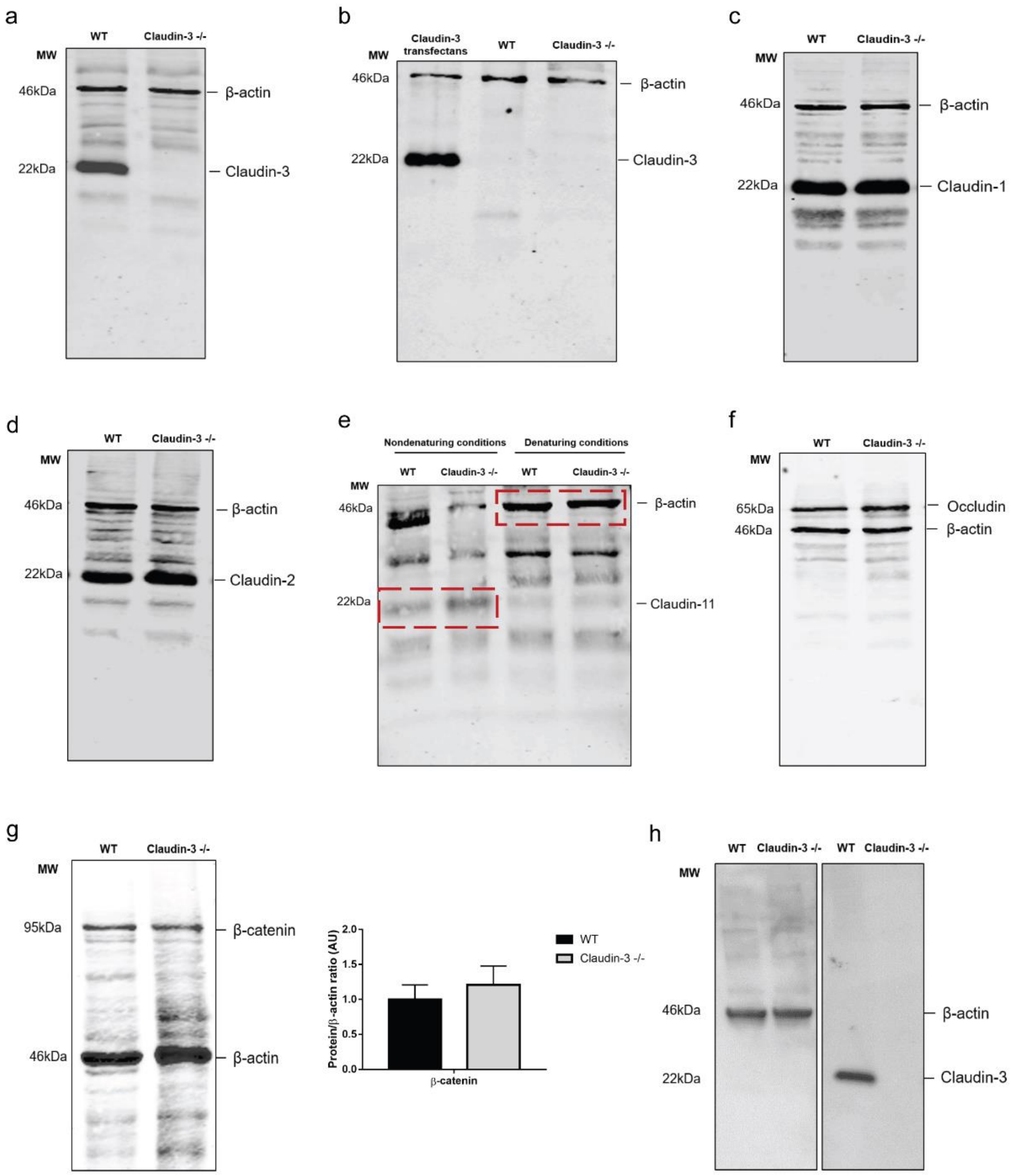

Supplementary Figure S7: Full length Western blots for claudin-1, claudin-2, claudin-3, claudin-11, β-catenin and occludin detection in samples of freshly isolated choroid plexuses, liver or brain microvessels pooled from 10 WT and claudin-3-/- C57BL/6J mice. Claudin-3 detection, with the Novus Biologicals antibody, in freshly isolated choroid plexuses, in freshly isolated brain microvessels and in liver is represented in **(a)** **(b)** and **(h),** respectively, while the cropped version of blots **(a)** and **(b)** can be found in Figure 1 and 3, respectively. Claudin-1, claudin-2, claudin-11, occludin and β-catenin detection in freshly isolated choroid plexuses are represented in **(c), (d), (e), (f)** and **(g),** respectively. The cropped version of these blots can be found in Figure 6. For claudin-11 detection **(e),** we ran the blot with choroid plexus samples from WT or claudin-3-/- C57BL/6J mice under nondenaturing conditions, since under denaturing conditions the signal for claudin-11 is lost, as demonstrated in the blot. For the detection of β-actin, the same samples were denatured prior loading into the gel, since under nondenaturing conditions, the signal for β-actin is lost. The considered bands for claudin-11 analysis are highlighted in red. For each protein, three independent choroid plexus/brain microvessel/liver samples from WT and claudin-3-/- C57BL/6J mice were analysed.

Supplementary Table S1: Claudin-3 genotype ratios of mice at weaning age

Claudin-3 genotype	Mice weaned		Female mice		Male mice	
	#	%	#	%	#	%
**WT**	560	27.3	266	13.0	294	14.3
**+/-**	1,009	49.2	495	24.1	514	25.1
**-/-**	481	23.5	227	11.1	254	12.4
**Total**	2,050	100	988	48.2	1,062	51.8

Supplementary Table S2: Observed cross-reactivity of commercially available anti-claudin-3 antibodies

Antigen	Company	Catalog/ Lot no	Species	Applications tested		
				**Western blots** **(lysates from claudin-1, claudin-3 and claudin-5 transfectants)**	**IF* staining** **(claudin-transfected HEK and L929 cells and pMBMECs)**	**IF staining** **(frozen brain sections)**
**Mouse Claudin-3 (amino acids 181- 219)**	**Abnova**	PAB270 35/ CJ36131	Rabbit poly-clonal IgG	**Used concentration: 2µg/mL**<br><br>**Observations:**<br>Detection of strong band at 22kDa in lysates of claudin-3 transfectants;<br><br>No detection of a band in lysates of claudin-1 and claudin-5 transfectants.	**Used concentration: 5µg/mL**<br><br>**Observations:**<br>Strong positive immunostaining of claudin-3 transfectants<br><br>Weak immunostaining of claudin-1 but not of claudin-5 and claudin-domain-containing-1 transfectants.	**Used concentration: 2,5µg/mL**<br><br>**Observations:**<br>WT mouse: Strong junctional staining of choroid plexus epithelium; positive staining of brain microvessels.<br><br>Claudin-3$^{-/-}$ mouse: No staining in choroid plexus; positive staining of brain microvessels.
**Mouse Claudin-3 (amino acidic sequence not stated)**	**Thermo Fisher Scientific**<br><br>**(Invitrogen)**	34-1700/ QA2119 19	Rabbit poly-clonal IgG	**Used concentration: 2µg/mL**<br><br>**Observations:**<br>Detection of strong band at 22kDa in lysates of claudin-3 transfectants;<br><br>No detection of a band in lysates of claudin-1 and claudin-5 transfectants.	**Used concentration: 5µg/mL**<br><br>**Observations:**<br>Strong positive immunostaining of claudin-3 transfectants<br><br>Weak immunostaining of claudin-1 but not of claudin-5 and claudin-domain-containing-1 transfectants.<br><br>Junctional staining of pMBMEC monolayers from WT and claudin-3$^{-/-}$ mice	**Used concentration: 2,5µg/mL**<br><br>**Observations:**<br>WT mouse: Strong junctional staining of choroid plexus epithelium; positive staining of brain microvessels.<br><br>Claudin-3$^{-/-}$ mouse: No staining in choroid plexus; positive staining of brain microvessels.
**Mouse Claudin-3 (amino acids 151- 200)**	**Aviva Systems Biology**	ARP428 31_P050 / QC1215 4-40526	Rabbit poly-clonal IgG	**Used concentration: 5µg/mL**<br><br>**Observations:**<br>Detection of strong band at 22kDa in lysates of claudin-3 transfectants;<br><br>No detection of a band in lysates of claudin-1 and claudin-5 transfectants	**Used concentration: 5µg/mL**<br><br>**Observations:**<br>Strong positive immunostaining of claudin-3 transfectants;<br><br>No immunostaining of claudin-1, claudin-5 and claudin-domain-containing-1 transfectants.	**Used concentration: 10µg/mL**<br><br>**Observations:**<br>WT mouse: Diffuse junctional staining of choroid plexus epithelium; no staining of brain microvessels.<br><br>Claudin-3$^{-/-}$ mouse: Diffuse junctional staining of choroid plexus epithelium; no staining of brain microvessels.

**Mouse Claudin-3 (full protein)**	**Proteintech**	16456-1-AP/ 00007923	Rabbit poly-clonal IgG	**Used concentration: 1.5μg/mL**<br><br>**Observations:** Detection of strong band at 22kDa in lysates of claudin-3 transfectants;<br><br>No detection of a band in lysates of claudin-1 and claudin-5 transfectants	**Used concentration: 1.5mg/mL**<br><br>**Observations:** Lack of immunostaining of claudin-3 transfectants<br><br>No immunostaining of claudin-1, claudin-5 and claudin-domain-containing-1 transfectants.	**Used concentration: 10μg/mL**<br><br>**Observations:** WT mouse: No junctional staining of choroid plexus epithelium and brain microvessels.<br><br>Claudin-$3^{-/-}$ mouse: No junctional staining of choroid plexus epithelium and brain microvessels.
**Mouse Claudin-3 (amino acidic sequence not stated)**	**Novus Biologicals**	NBP1-35668/ 160716LVC	Rabbit poly-clonal IgG	**n.d.§**	**Used concentration: 1:50**<br><br>**Observations:** Junctional staining of pMBMECs from WT and claudin-$3^{-/-}$ mice.	**Used concentration: 1:50**<br><br>**Observations:** WT mouse: Diffuse junctional staining of choroid plexus epithelium; no staining of brain microvessels.<br><br>Claudin-$3^{-/-}$ mouse: No junctional staining of choroid plexus epithelium and no staining of brain microvessels.

*IF = immunofluorescence; §n.d. = not done

Supplementary Table S3: Expression of selected cell genes by qPCR

Gene of interest	Brain microvessels WT	Brain microvessels (claudin-3$^{-/-}$)	pMCPECs WT	pMCPECs (claudin-3$^{-/-}$)	Choroid Plexus WT	Choroid Plexus (claudin-3$^{-/-}$)
**Claudin-1**	n.d.*	n.d.	25.40 ±1.36	20.71±1.58	26.56±2.58	26.50±1.56
**Claudin-2**	n.d.	n.d.	27.46±1.12	27.53±2.49	28.26±1.82	25.80±1.36
**Claudin-3**	n.d.	n.d.	30.05±1.3	n.d.	28.39±1.31	n.d.
**Claudin-5**	21.92±1.02	22.56±1.05	n.d.	n.d.	30.26±1.50	30.29±1.60
**Claudin-11**	n.d.	n.d.	32,13±0.95	25.66±2.07	31.22±1.80	29.52±2.21
**Occludin**	31.13±2.31	32.02±1.85	32.04±1.38	32,54±1.96	29.69±2.76	29.44±2.80
**Transthyretin**	27.46±1.37	29.68±2.71	19.41±052	18.50±1.33	14.61±1.16	14.64±1.25
**E-cadherin**	n.d.	n.d.	n.i.§	n.i.	n.i.	n.i.
**S16**	25.60±1.57	26.91±0.89	24.64±1.91	24.04±1.32	24.59±2.04	24.62±1.69

*n.d. – not detectable

§n.i. – not included

Supplementary Table S4: Sequence of primers used for the qPCR analysis

Target mRNA	Sequence
S16 ribosomal protein (control)	sense: GATATTCGGGTCCGTGTGA<br>antisense: TTGAGATGGACTGTCGGATG
Claudin-1	sense: ACTCCTTGCTGAATCTGAACAGT<br>antisense: GGACACAAAGATTGCGATCAG
Claudin-2	sense: TGTGAATGAACTGAAGGAAAGC<br>antisense: ATCCTGCACCCAGCTGTATT
Claudin-3	sense: CGTACAAGACGAGACGGCCAAG<br>antisense: CACGTACAACCCAGCTCCCATC
Claudin-5	sense: ACGGGAGGAGCGCTTTAC<br>antisense: GTTGGCGAACCAGCAGAG
Claudin-11	sense: TGGAGTGGCCAAGTACAGG<br>antisense: GACAATGGCGCAGAGAGC
Occludin	sense:<br>GGTCTCTACGTGGATCAATATTTGTA<br>antisense: AACCCCAGGACAATGGCTA
Transthyretin	sense: CATGAATTCGCGGATGTG<br>antisense: GATGGTGTAGTGGCGATGG
VE-cadherin	sense: GTGATGTTGGCGGTGTTGT<br>antisense: GTTCAAGTTTGGCCTGAAGAA
E-cadherin	sense: CAGAATGACAACAGGCCAGA<br>antisense: CATTAGCAGCTGGCTGAGAGA

Supplementary Table S5: List of antibodies used in this study

Primary antibodies							
Antigen	Company	Catalog no.	Lot no.	Host species	Concentration		
					Western blot	IF* staining brain cryosections	IF staining tissue culture
Mouse Claudin-1	Thermo Fisher Scientific (Invitrogen)	51-9000	1364487A	Rabbit polyclonal IgG	1.25 µg/mL	n.d.$	1.25 µg/mL
Mouse Claudin-2	Thermo Fisher Scientific (Invitrogen)	51-6100	QE215499	Rabbit polyclonal IgG	1.25 µg/mL	n.d.	1.25 µg/mL
Mouse Claudin-3	Novus Biologicals	NBP1-35668	160716LVC	Rabbit polyclonal IgG	1:1000	1:50	1:50
Mouse Claudin-3	Thermo Fisher Scientific (Invitrogen)	34-1700	QA211919	Rabbit polyclonal IgG	n.d.	n.d.	1.25 µg/mL
Mouse Claudin-5	Thermo Fisher Scientific (Invitrogen)	34-1600	1105603A	Rabbit polyclonal IgG	1.25 µg/mL	n.d.	1.25 µg/mL
Mouse OSP/Claudin-11	Thermo Fisher Scientific (Invitrogen)	36-4500	919259A	Rabbit polyclonal IgG	1.25 µg/mL	n.d.	1.25 µg/mL
Mouse Occludin	Thermo Fisher Scientific (Invitrogen)	71-1500	RG235307	Rabbit polyclonal IgG	1.25 µg/mL	n.d.	1.25 µg/mL
Mouse β-actin	Merck	A5316	052M4793	Mouse monoclonal IgG2a	1:3,000	n.d.	n.d.
Mouse β-catenin	BD Transduction Laboratories™	610154	2300995	Mouse monoclonal IgG1	1:1,000	n.d.	1.25 µg/mL
Mouse ZO-1	Thermo Fisher Scientific (Invitrogen)	61-7300	QG215368	Rabbit polyclonal IgG	n.d.	n.d.	1.25 µg/mL
Mouse ZO-2	Thermo Fisher Scientific (Invitrogen)	71-1400	389780A	Rabbit polyclonal IgG	n.d.	n.d.	1.25 µg/mL
Mouse pan-Cytokeratin	Merck	C2562	061M4752	Mouse monoclonal C-11+PCK-26+CY-90+KS-1A3+M20+A53-B/A2 (mixture)	n.d.	n.d.	1:100

Mouse PECAM-1	In house	Clone Mec13.3	n.a.§	Monoclonal rat IgG2a	n.d.	n.d.	20 µg/mL
Mouse JAM-A	In house	Clone BV12	n.a.	Monoclonal rat IgG2a	n.d.	n.d.	20 µg/mL
Mouse VE-cadherin	In house	Clone 11D4	n.a.	Monoclonal rat IgG	n.d.	n.d.	20 µg/mL
Mouse Fibronectin	Agilent Technologies (DAKO)	A0245	097	Rabbit polyclonal IgG	n.d.	n.d.	12.5 µg/mL
**Secondary antibodies**							
**Antigen**	**Company**	**Catalog no.**	**Lot no.**	**Antibody/ reagent**	**Concentration**		
					**Western blot**	**IF staining brain cryosections**	**IF staining tissue culture**
Rabbit IgG (H+L)	Thermo Fisher Scientific (Invitrogen)	A21109	1716991	Goat polyclonal IgG Alexa Fluor® 680	1:10,000	n.d.	n.d.
Mouse IgG (H+L)	Rockland Immunochemicals	605-732-125	17583	Goat polyclonal IgG IRDye® TM 800	1:10,000	n.d.	n.d.
Mouse IgG (H+L)	Jackson ImmunoResearch	111-165-144	94787	Donkey polyclonal IgG Cy3	n.d.	1:200	2.5µg/mL
Rabbit IgG (H+L)	Thermo Fisher Scientific (Invitrogen)	A11034	1616933	Goat polyclonal IgG Alexa Fluor® 488	n.d.	n.d.	10µg/mL
Rat IgG (H+L)	Thermo Fisher Scientific (Invitrogen)	A11006	1214851	Goat polyclonal IgG Alexa Fluor® 488	n.d.	1:200	n.d.
n.a.	Biolegend	405235	-	Alexa Fluor® 488 Streptavidin	n.d.	1:200	n.d.
Mouse IgG	Vector	MKB-225	ZA0728	Mouse (M.O.M ™) Biotinylated	n.d.	n.d.	1:100

*IF – immunofluorescence; $ n.d. – not done; §n.a. – not applicable
